# Supplementary material for: A microRNA-initiated DNAzyme motor operating in living cells
Source: Nat Commun. 2017 Mar 6;8:14378. doi: 10.1038/ncomms14378 (PMC5343503; doi:10.1038/ncomms14378)
Supplement: Supplementary Information — Supplementary Figures and Supplementary Tables. [file ncomms14378-s1.pdf]

**Supplementary Table 1.** Oligonucleotide sequences used in the study

| **Oligonucleotides** | **Sequences (5' → 3' )** |
|---|---|
| Substrate strand | HS-(T)$_{14}$CACTATrAGGAAGAGAT-6-Carboxyfluorescein (FAM) |
| Lock -4 | GAGACACAAATTCGGTTCTACAGGGTA |
| Lock -5 | AGAGACACAAATTCGGTTCTA CAGGGTA |
| Lock -6 | AAGAGACACAAATTCGGTTCTACAGGGTA-Cy5 |
| Lock -7 | GAAGAGACACAAATTCGGTTCTACAGGGTA |
| Free control DNAzyme | ATCTCTTCTCCGAGCCGGTCGAAATAGTGAA<br>Arm1 Catalytic core Arm2 |
| DNAzyme strand linked to AuNP | HS-(T)$_{42}$TAGAACCGAATTTGTGTCTCTTCTCCGAGCCGGTCGAAATAGT<br>Arm1 Catalytic core Arm2 |
| Mutant DNAzyme strand linked to AuNP | HS-(T)$_{42}$TAGAACCGAATTTGTGTCTCTTCTCCGATCCGGTCTAAATAGT |
| Target microRNA-10b | UACCCUGUAGAACCGAAUUUGUG |
| DNA Target | TACCCTGTAGAACCGAATTTGTG |
| Mismatch-1 | TACACTGTAGAACCGAATTTGTG |
| Mismatch-2 | TACCATGTAGAACCGAATTTGTG |
| Mismatch-3 | TACCCTGTAGAAGCGAATTTGTG |
| Mismatch-4 | TACCCTGTAGAACCTAATTTGTG |
| Mismatch-5 | TACCCTGTAGAACCGAATTTTTG |
| Biotin-DNAzyme 8-17E | Biotin-TTTTTTTTTTTTTTTTTTTTTTTTTTTTTTTTTTTTTTTTTTTTTTG<br>TCTCTTCTCCGAGCCGGTCGAAATAGT |
| Biotin-DNAzyme 8-17 | Biotin-TTTTTTTTTTTTTTTTTTTTTTTTTTTTTTTTTTTTTTTTTTTTTTG<br>TCTCTTCTCCGAGCCGGACGAATAGT |
| Biotin-DNAzyme 10-23 | Biotin-TTTTTTTTTTTTTTTTTTTTTTTTTTTTTTTTTTTTTTTTTTTTTTTT<br>TCTCTTCAGGCTAGCTACAACGATAGT |
| Poly(T)-biotin | HS-TTTTTTTTTTTTTTTTTTTTTTTTTTTTTT-Biotin |
| Substrate strand for DNAzyme 10-23 | HS-TTTTTTTTTTTTTTACTAT rGrU GAAGAGAT-FAM |

**Supplementary Table 2**. Selection factor of the DNAzyme motor for five variants of single-base mismatch.

| Mismatch variants | 1 | 2 | 3 | 4 | 5 |
|---|---|---|---|---|---|
| Selection factor | 5.1 | 8.8 | 5.2 | 16.7 | 6.8 |

The selection factor was calculated by using the following equation [Supplementary Equation (1)]:

$$\text{Selection factor} = \frac{F_{[\text{Target}]} - F_{[\text{Control}]}}{F_{[\text{Mismatch}]} - F_{[\text{Control}]}} \qquad (1)$$

**Supplementary Table 3.** Oligonucleotide sequences used to construct the DNAzyme motor for tracing the operation of the DNAzyme motor on individual AuNPs

| **Oligonucleotides** | **Sequences (5' → 3' )** |
|---|---|
| Signal reporter | Cy5- TCT GTG ACG TAC CTT CTC TGA TCA TCC TGT TT-HS |
| Substrate strand | ACAGGATGATCAGAGAAGGTACGTCACAGA TCTCACTATrAGGAAGAGAT-/5IAbRQ/ |
| Lock-6 | AAGAGACACAAATTCGGTTCTACAGGGTA |
| DNAzyme strand | HS-(T)$_{43}$AGAACCGAATTTGTGTCTCTTCTCCGAGCCGGTCGAAATAGT<br>Arm1 Catalytic core Arm2 |
| Target microRNA-10b | UACCCUGUAGAACCGAAUUUGUG |
| Target DNA | TACCCTGTAGAACCGAATTTGTG |

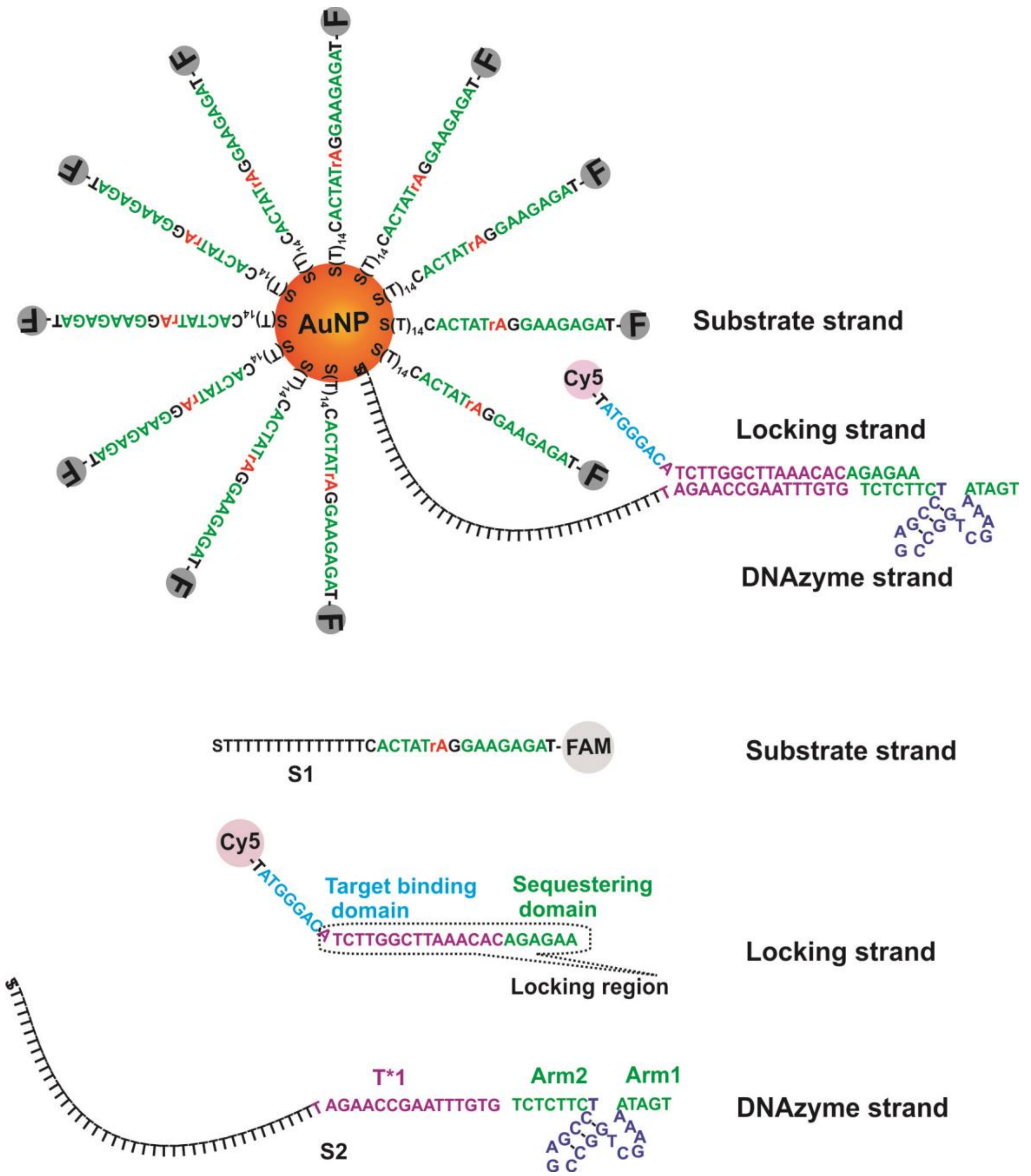


**Supplementary Figure 1.** Design and sequences used in the miRNA-initiated DNAzyme motor. The complete motor system is a functionalized AuNP onto which are conjugated hundreds of substrate strands and dozens of locked DNAzyme strands. The substrate strand is a DNA-RNA chimeric sequence composed of an RNA nucleotide flanked by two DNA domains complementary to **Arm 1** and **Arm 2** of the DNAzyme. To enhance the accessibility of the substrate strand to the DNAzyme, a 14-thymine spacer **S1** is added to the substrate at the 5′-end that is conjugated to AuNP. The 3′-end of the substrate is labeled with a FAM molecule. The fluorescence of the FAM molecules on the substrate strands is quenched by the AuNP. The DNAzyme is extended to include a single-stranded spacer **S2** linked to the 3′-end of **Arm 2**. The spacer **S2** comprises a 42-thymine domain that is conjugated to AuNP and a 16-nt domain **T*1**. **T*1** and **Arm 2** of the DNAzyme strand form the locking region. A DNA locking strand is designed according to the target sequence and the DNAzyme strand. The locking strand contains a target-binding domain complementary to the target miRNA (miR-10b) and a 6-nt sequestering domain complementary to **Arm 2** of the DNAzyme. The hybridization of the locking strand to the domain **T*1** and **Arm 2** forms a duplex with a 7-nt toehold at the 3′-end of the locking strand, which sequesters **Arm 2** and prevents it from binding to the substrate strands, making the DNAzyme motor inactive.

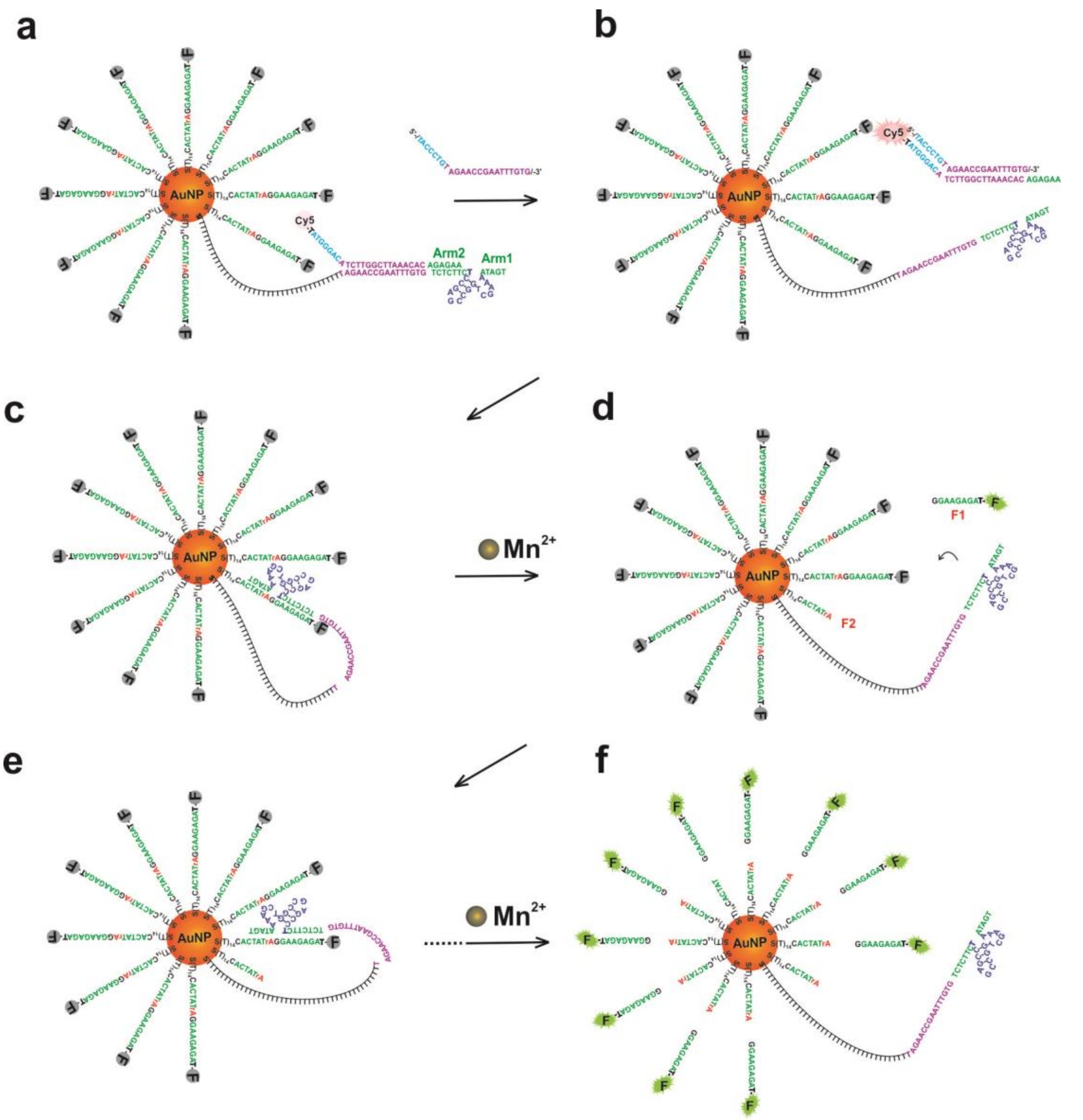


**Supplementary Figure 2.** The operation of the miRNA-initiated DNAzyme motor. In the absence of the target miRNA, the activity of the DNAzyme motor is locked by the locking strand and the motor is not operational (a). Upon addition of the target miRNA, the target miRNA hybridizes to the locking strand and releases it from the DNAzyme through a toehold-mediated strand displacement reaction, exposing the sequestered **Arm 2** of the DNAzyme motor. The strand displacement reaction forms a duplex between the locking strand and target miRNA, making the locking strand stay away from the AuNP surface. Thus, the fluorescence of the Cy5 molecule in the duplex is restored, which is used to signal the location of target miRNA (b). Meanwhile, the liberated DNAzyme motor hybridizes with a substrate strand on the AuNP (c). In the presence of cofactor $Mn^{2+}$, DNAzyme is activated to cleave the substrate at the single-ribonucleotide junction, generating two DNA segments **F1** and **F2**. FAM-containing **F1** dissociates from **Arm 2** and leaves the AuNP surface, restoring the fluorescence of the FAM molecule (d). Meanwhile, the DNAzyme dissociates from the **F2** and subsequently hybridizes to the next substrate strand, achieving the walking of the motor from one substrate strand to the next (e). This stepwise walking is repeated autonomously, driving the DNAzyme motor to traverse along the AuNP surface (f).

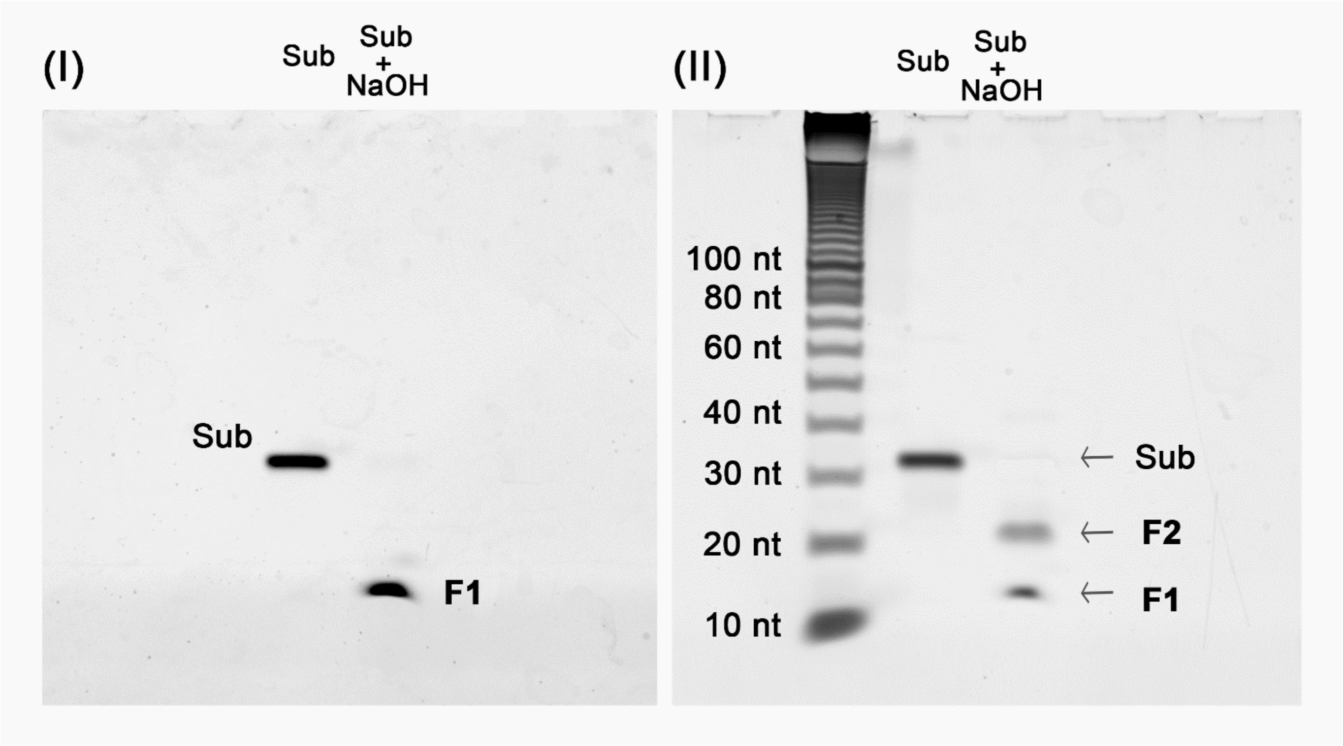


**Supplementary Figure 3a**. Gel images showing FAM-containing substrate strand and its hydrolysis products, **F1** and **F2.** The same gel was imaged first by detecting the FAM label (Image I) and then by SYBR Gold staining (Image II). Only bands corresponding to the substrate strand (**Sub**) and **F1** are detectable (Image I) because they contain FAM label. The bands corresponding to 10 nt DNA ladder, substrate strand, **F1** and **F2** were all detectable after SYBR Gold staining (Image II).

The substrate strand (Sub, 30 nt) and its hydrolysis products **F1** (9 nt) and **F2** (21 nt) were separated on 10% denaturing PAGE gel. The gel was first imaged by detection of FAM (Image I). Only the FAM-containing DNA strands, i.e., the substrate strand and **F1,** were detectable. The gel was then imaged by SYBR Gold staining, all DNA, including the 10 nt DNA ladder, substrate strand, **F1,** and **F2,** were detected (Image II). The migration of the substrate strand and **F1** were slightly lower than that of the corresponding DNA strands of 30 nt and 10 nt of DNA ladder, suggesting that the presence of FAM label reduced the migration of the substrate strand and **F1**. Because SYBR Gold staining resulted in a much lower intensity than FAM detection for the **F1** band, the subsequent gel electrophoresis experiments used FAM detection, as shown in Supplementary Figures 3b, 5, 6, and 7. The FAM-containing substrate strand (**Sub**) and its hydrolysis product (**F1**) served as DNA markers. The hydrolysis products **F1** and **F2** were obtained by hydrolysis of the single ribonucleotide bond of the substrate using 2 M NaOH.

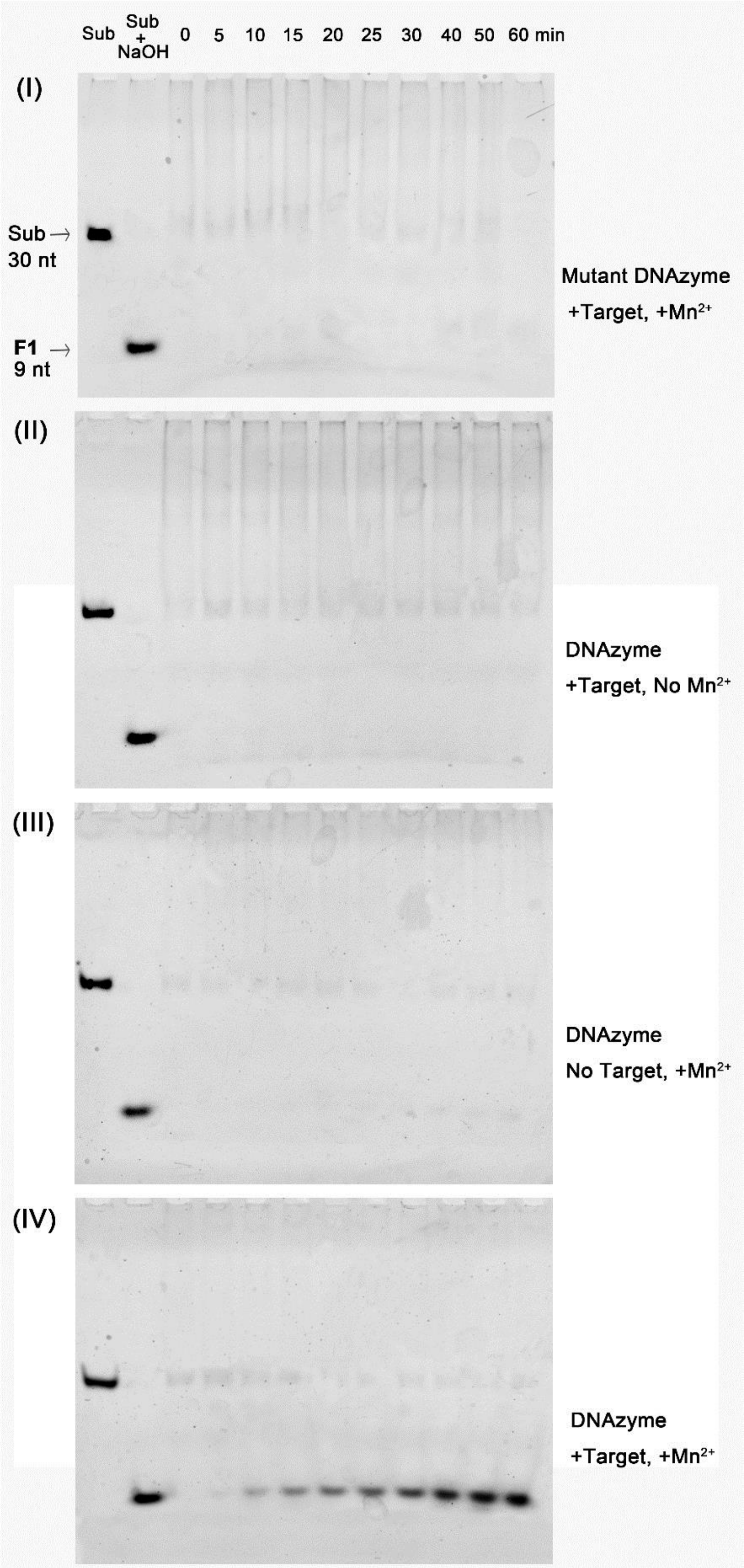


**Supplementary Figure 3b.**

**Supplementary Figure 3b.** Gel images showing the expected fluorescent substrate segment **F1** cleaved by the DNAzyme motor (Image IV), no cleavage of the fluorescent substrate by a mutant DNAzyme motor (Image I), and no cleavage of the fluorescent substrate by the DNAzyme motor in the absence of the target or the cofactor $Mn^{2+}$ (Images II and III).

The DNAzyme or mutant DNAzyme motor system at a concentration equivalent to 7 nM AuNPs was mixed with 16 nM target DNA sequence in the autoclaved buffer (pH 8.0), containing 25 mM Tris-acetate and 200 mM NaCl. After incubation at room temperature for 20 min, 0.5 mM $Mn^{2+}$ was added. Following the addition of $Mn^{2+}$ (referred to as time 0), 5 μL of the reaction solution was sampled repeatedly, to which 5 μL of 50 mM EDTA was added to chelate the cofactor $Mn^{2+}$ and thus stop the catalytic reaction. The reaction solution was then analyzed by gel electrophoresis. The far left lane is the full-length FAM-substrate in the autoclaved buffer. No **F1** is detectable, suggesting that the substrate is stable. The second lane from left is the control showing the fluorescent segment **F1** obtained by hydrolysis of the single ribonucleotide bond of the substrate using 2 M NaOH.

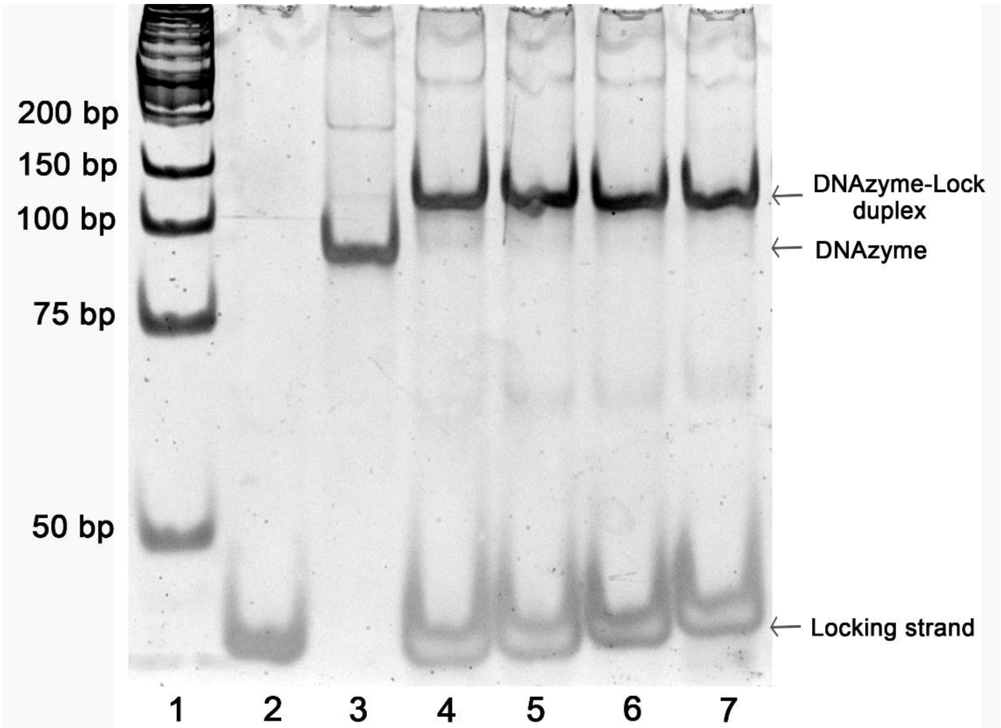


**Supplementary Figure 4**. Gel images showing the formation of the duplexes between the DNAzyme strand and four different locking strands. Lane 1: DNA ladder from 25 nt to 766 nt; lane 2: Lock-4 (27 nt); lane 3: DNAzyme strand (85 nt); lane 4: mixture of the DNAzyme strand and Lock-4 at 1:3 molar ratio; lane 5: mixture of the DNAzyme strand and Lock-5 at 1:3 molar ratio; lane 6: mixture of the DNAzyme strand and Lock-6 at 1:3 molar ratio; lane 7: mixture of the DNAzyme strand and Lock-7 at 1:3 molar ratio. In lanes 4, 5, 6, and 7, the darkest band corresponds to the duplexes formed between the DNAzyme strand and the locking strand, Lock-4, Lock-5, Lock-6, and Lock-7, respectively. The lengths of Lock-4, Lock-5, Lock-6, and Lock-7 are 27 nt, 28 nt, 29 nt, and 30 nt, respectively. With the use of Lock-6 and Lock-7, no free DNAzyme is visible from the gel (lanes 6 and 7), suggesting complete formation of the duplexes between the DNAzyme and either Lock-6 or Lock-7.

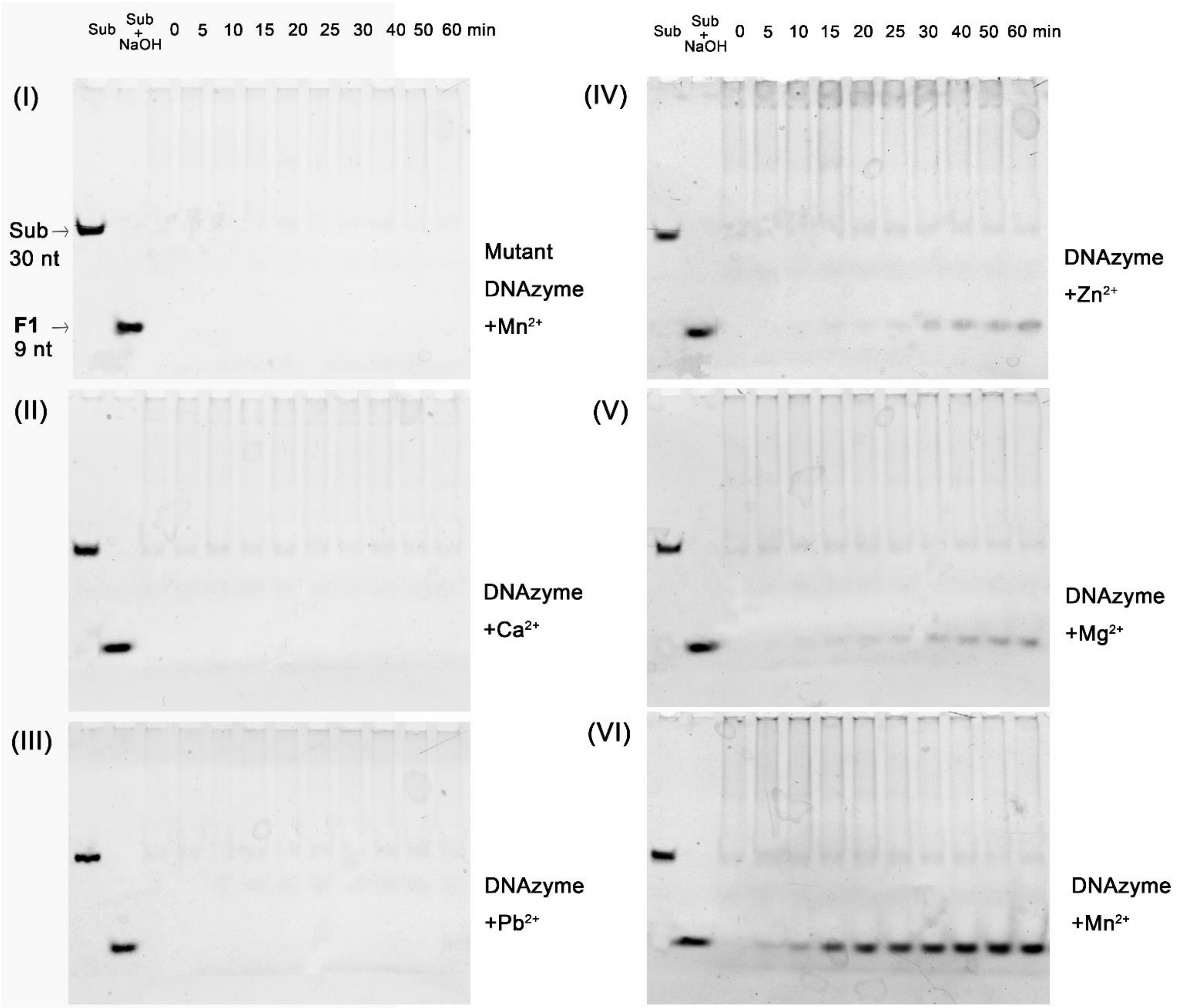


**Supplementary Figure 5.** Gel images showing the expected fluorescent segment **F1** from cleavage of the substrate by the DNAzyme motor in the presence of cofactor $Mn^{2+}$ (Image VI), no cleavage of the fluorescent substrate by a mutant DNAzyme motor (Image I), and various degrees of cleavage of the fluorescent substrate by the DNAzyme motor in the presence of $Ca^{2+}$, $Pb^{2+}$, $Zn^{2+}$, or $Mg^{2+}$, as alternative cofactors (Images II-V).

The DNAzyme or mutant DNAzyme motor system at a concentration equivalent to 7 nM AuNPs was mixed with 16 nM target DNA sequence in autoclaved buffer (pH 8.0), containing 25 mM Tris-acetate and 200 mM NaCl. After incubation at room temperature for 20 min, 0.5 mM $Mn^{2+}$, 10 mM $Ca^{2+}$, 0.2 mM $Pb^{2+}$, 0.01 mM $Zn^{2+}$, or 10 mM $Mg^{2+}$ was added. Following the addition of cofactor (referred to as time 0), 5 µL of the reaction solution was sampled repeatedly, to which 5 µL of 50 mM EDTA was added to chelate the cofactor ion and thus stop the catalytic reaction. The solution was then analyzed by gel electrophoresis. The far left lane is the FAM-substrate in the autoclaved buffer. No cleavage product (**F1**) is detectable, suggesting that the substrate is stable. The second lane from left is the control showing the fluorescent substrate segment **F1** obtained by hydrolysis of the ribonucleotide bond of the substrate using 2M NaOH.

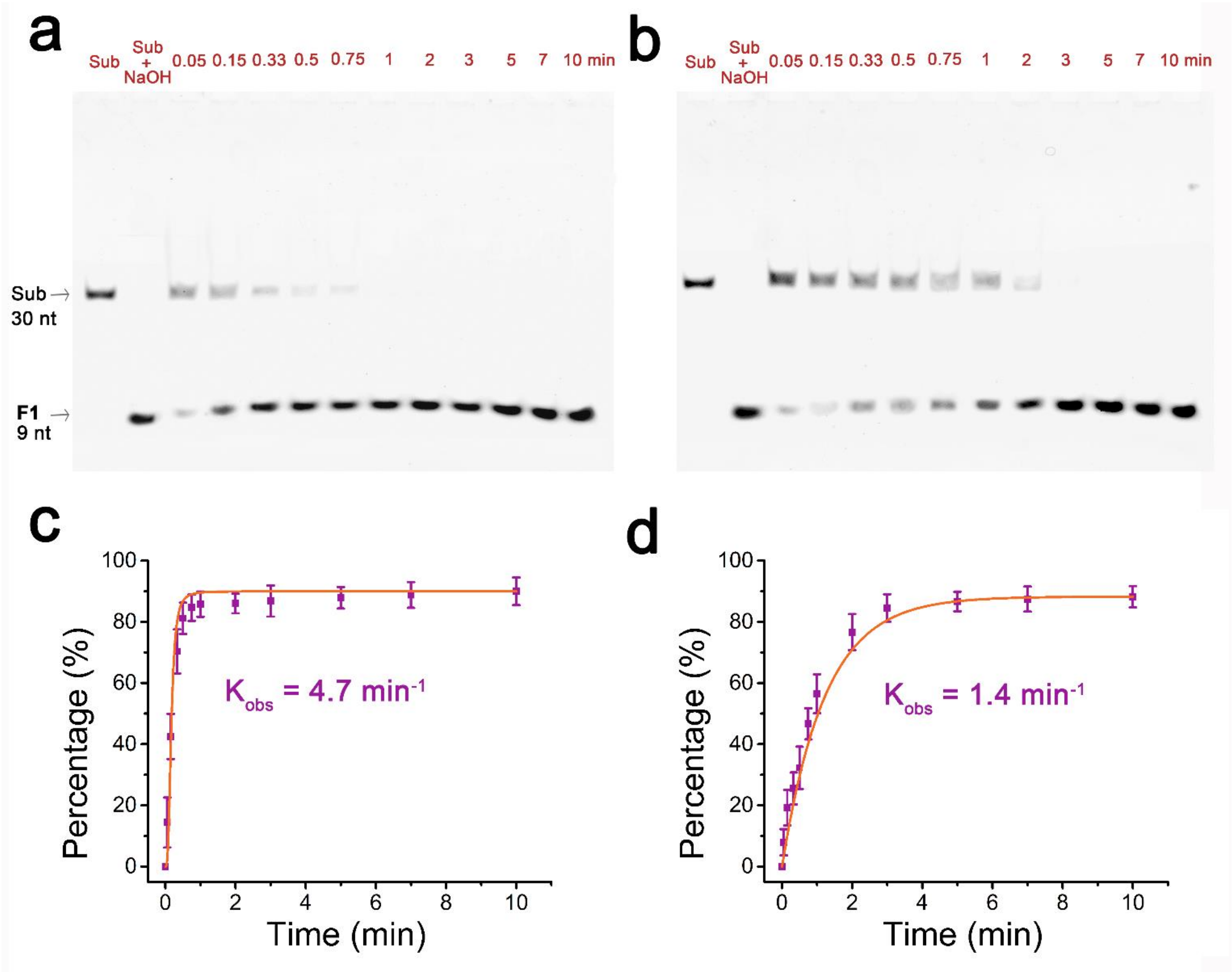


**Supplementary Figure 6.** Comparison of single-turnover cleavage rate of the substrate by the DNAzyme motor tested using either $Mn^{2+}$ or $Mg^{2+}$ as the cofactor. (a) Gel images showing the single-turnover cleavage of the substrate by the DNAzyme in the presence of 0.5 mM $Mn^{2+}$. (b) Gel images showing the single-turnover cleavage of the substrate by the DNAzyme in the presence of 10 mM $Mg^{2+}$. (c) Curve fitting showing a single-turnover cleavage rate ($K_{obs}$) of 4.7 $min^{-1}$ for the DNAzyme with 0.5 mM $Mn^{2+}$ as the cofactor. (d) Curve fitting showing a single-turnover cleavage rate ($K_{obs}$) of 1.4 $min^{-1}$ for the DNAzyme with 10 mM $Mg^{2+}$ as the cofactor. Error bars represent one standard deviation from duplicate experiments.

10 µM of the free control DNAzyme (sequence in Supplementary Table 1, containing an 8-nt **Arm 1** and an 8-nt **Arm 2**) was mixed with 1 µM FAM-labeled substrate autoclaved buffer (pH 8.0) containing 25 mM Tris-acetate and 200 mM NaCl. After incubation for 10 min, 0.5 mM $Mn^{2+}$ or 10 mM $Mg^{2+}$ was added to initiate the catalytic cleavage of the substrate by the DNAzyme. From 0.05 min to 10 min following the addition of either $Mn^{2+}$ or $Mg^{2+}$, 10 µL of reaction solution was sampled, to which 10 µL of 50 mM EDTA and 8 M urea was added. The solution was subjected to PAGE analysis.

The intensity of the bands in the gel images was measured using ImageJ 1.47. The sum of the intensity of the substrate band and the product band in the first lane at 0.05 min was used to represent the total amount of the fluorescent substrate and product, serving as the denominator in the calculation of the percentage cleaved. The intensity of the cleaved product band in each lane from 0.05 to 10 min was used as the numerator in the calculation of the percentage cleaved. In the gel images of (a) and (b), the far left lane is the FAM-substrate in the autoclaved buffer. No cleavage product is detectable, suggesting that the substrate is stable. The second lane from left is the control showing the fluorescent segment **F1** obtained by hydrolysis of the ribonucleotide bond of the substrate using 2M NaOH.

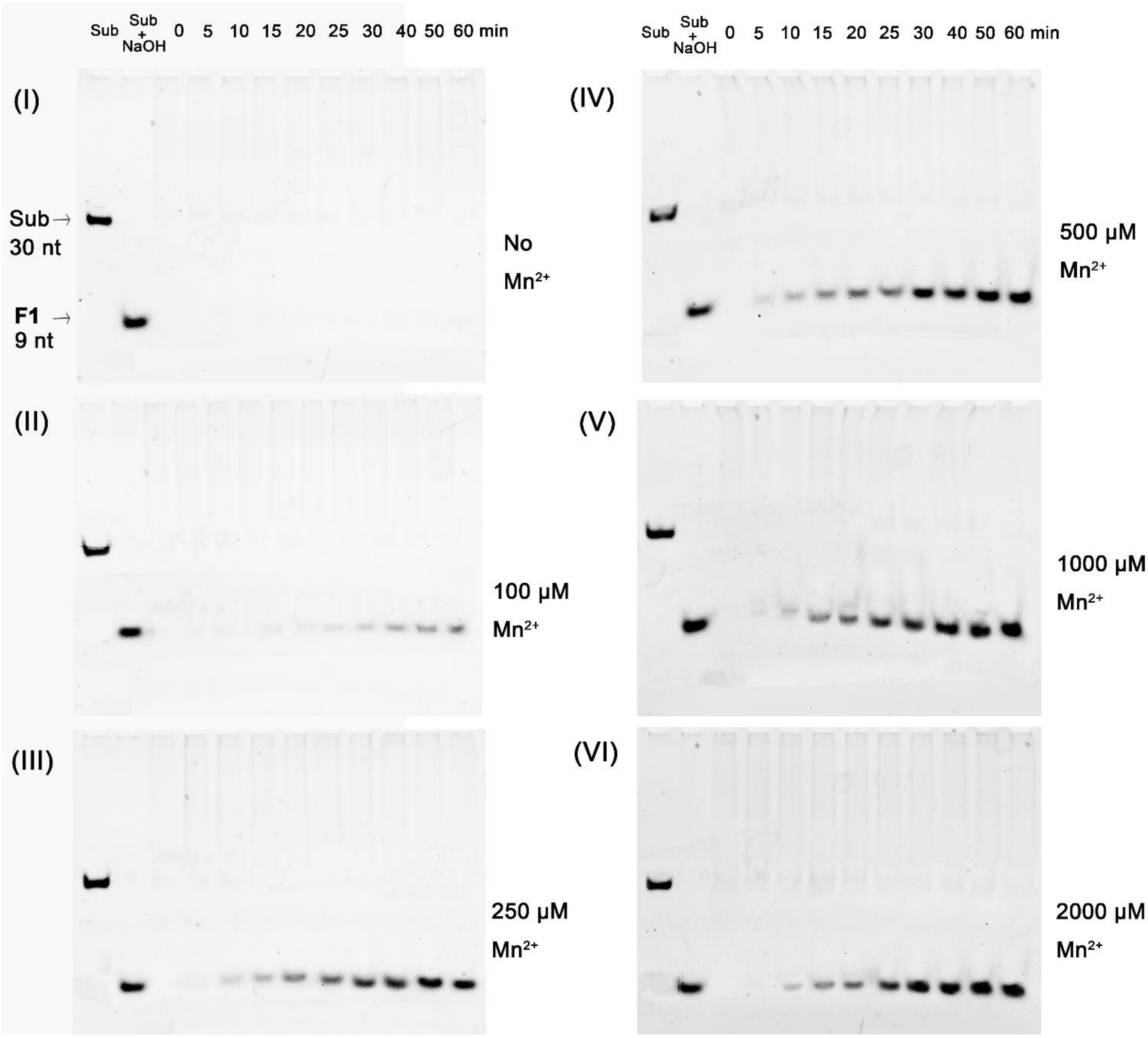


**Supplementary Figure 7.** Gel images showing no cleavage of the fluorescent substrate in the absence of cofactor $Mn^{2+}$ (Image I) and the various amounts of the fluorescent substrate segment **F1** cleaved by the DNAzyme motor in the presence of different concentrations of the cofactor $Mn^{2+}$ (Images II-VI).

The DNAzyme or mutant DNAzyme motor system at a concentration equivalent to 7 nM AuNPs was mixed with 16 nM target DNA sequence in autoclaved buffer (pH 8.0) containing 25 mM Tris-acetate and 200 mM NaCl. After incubation at room temperature for 20 min, 0, 100, 250, 500, 1000, or 2000 μM $Mn^{2+}$ was added to initiate the catalytic cleavage of the substrate. From 0 min to 60 min after the addition of $Mn^{2+}$, 5 μL of the reaction solution was repeatedly sampled, to which 5 μL of 50 mM EDTA was added to chelate the cofactor $Mn^{2+}$ and thus stop the catalytic reaction. The reaction solution was analyzed by gel electrophoresis. The far left lane is the FAM-substrate in the autoclaved buffer. No cleavage product is detectable, suggesting that the substrate is stable in the autoclaved buffer. The second lane from left is the control showing the fluorescent substrate segment **F1** obtained by hydrolysis of the ribonucleotide bond of the substrate using 2M NaOH.

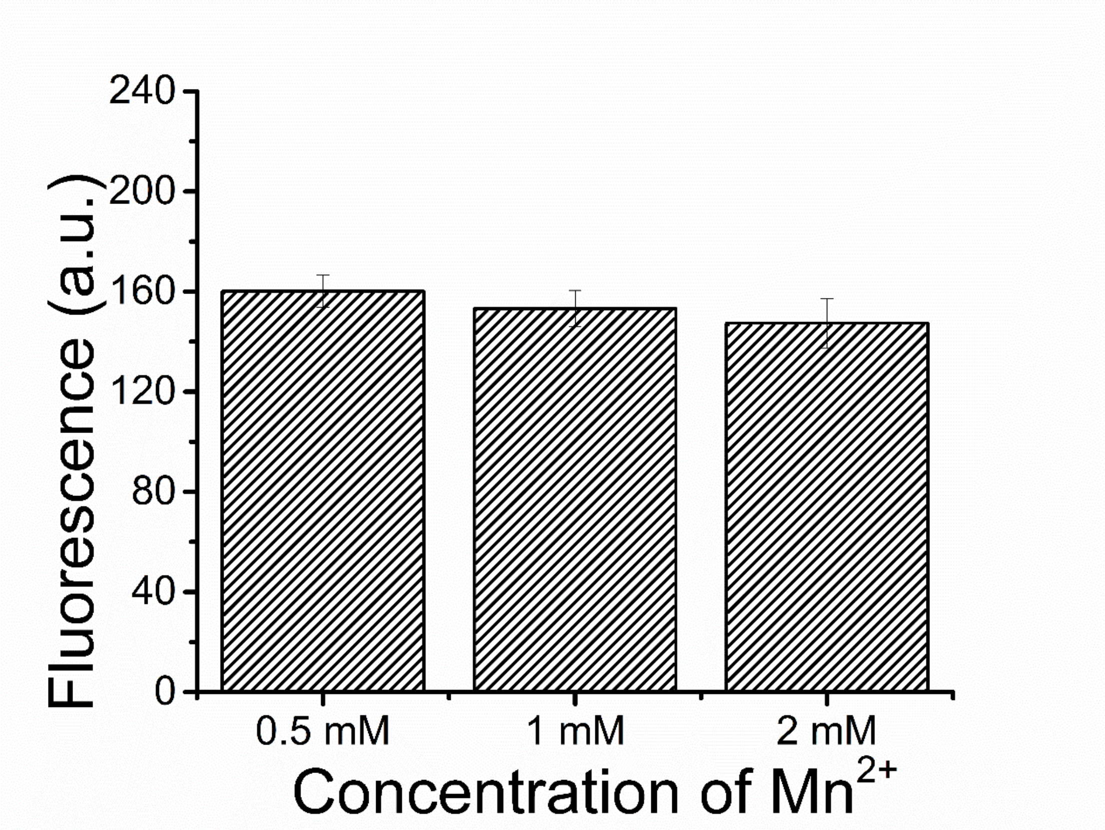


**Supplementary Figure 8.** Effect of $Mn^{2+}$ concentration on the fluorescence intensity of FAM-labeled substrate. The concentration of the FAM-labeled substrate is 10 nM. Error bars represent one standard deviation from triplicate experiments.

In a comparison of the fluorescence generated by the DNAzyme motor that was initiated by 200 pM target DNA and activated by different concentrations of the cofactor $Mn^{2+}$ (Fig. 3b), the fluorescence intensity from the use of 1 mM and 2 mM of $Mn^{2+}$ was slightly lower than that from the use of 0.5 mM $Mn^{2+}$ (Fig. 3b). The slightly lower fluorescence intensity of the fluorescent substrate segment **F1** is probably because of a combination of the following two reasons: the higher $Mn^{2+}$ concentration decreased the cleavage rate (Supplementary Fig. 7) by slowing down the dissociation of DNAzyme from the substrate segment **F2**; and a reduced fluorescence intensity of **F1** due to fluorescence quenching (Supplementary Fig. 8).

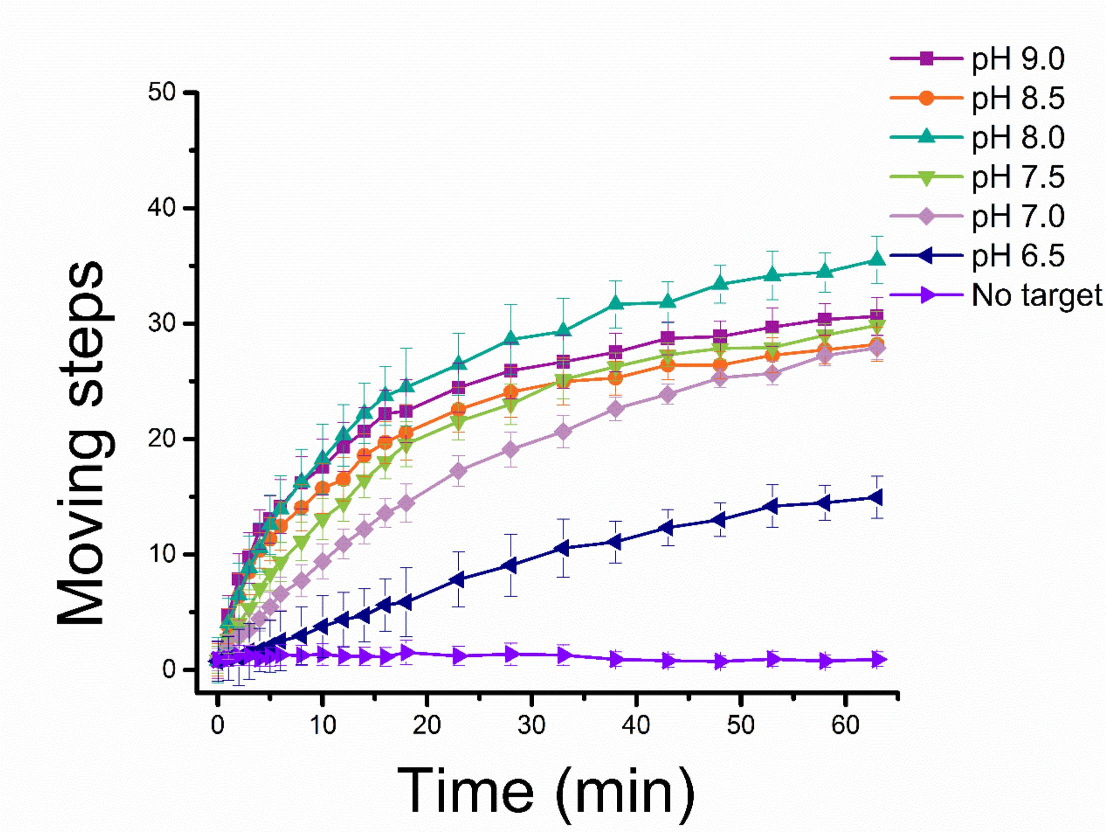


**Supplementary Figure 9**. Operating curves of the DNAzyme motor under different pH conditions. The concentration of $Mn^{2+}$ is 500 μM, and the concentration of the DNA target is 200 pM (or 0 pM for the control). Error bars represent one standard deviation from duplicate experiments. Note that at higher pH (e.g., >8.5), $Mn^{2+}$ could precipitate as $Mn(OH)_2$, according to $K_{sp}$ of $Mn(OH)_2$ which is $1.9\times10^{-13}$.

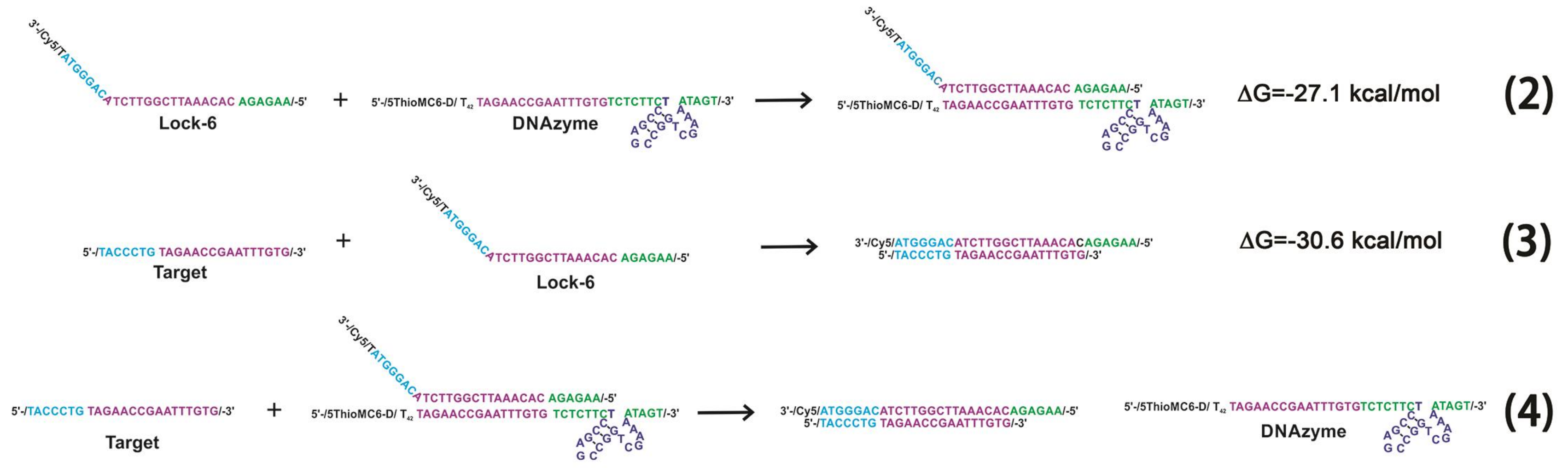


**Supplementary Figure 10**. Schematics showing the pertinent hybridization reactions. Supplementary Equation (2) illustrates hybridization between Lock-6 and DNAzyme strand; Supplementary Equation (3) illustrates hybridization reaction between Lock-6 and the target DNA; Supplementary Equation (4) illustrates toehold-mediated strand exchange reaction between the target DNA and the duplex of Lock-6 and DNAzyme strand.

The design of the locking strand that contains a sequestering domain in addition to the target-binding domain contributes to high specificity of the DNAzyme motor for the target. The Lock-6 has a 7-nt toehold to ensure highly efficient strand displacement and a 6-nt sequestering domain that is first bound and then exposed after the strand displacement reaction, reducing the ΔG of the reaction and thereby improving the specificity. The ΔG of hybridization between Lock-6 and the target miRNA is -30.6 kcal $mol^{-1}$, and that between Lock-6 and the DNAzyme strand is -27.1 kcal $mol^{-1}$. Thus, the ΔG of strand displacement reaction is only -3.5 kcal $mol^{-1}$, leading to the high specificity of the reaction

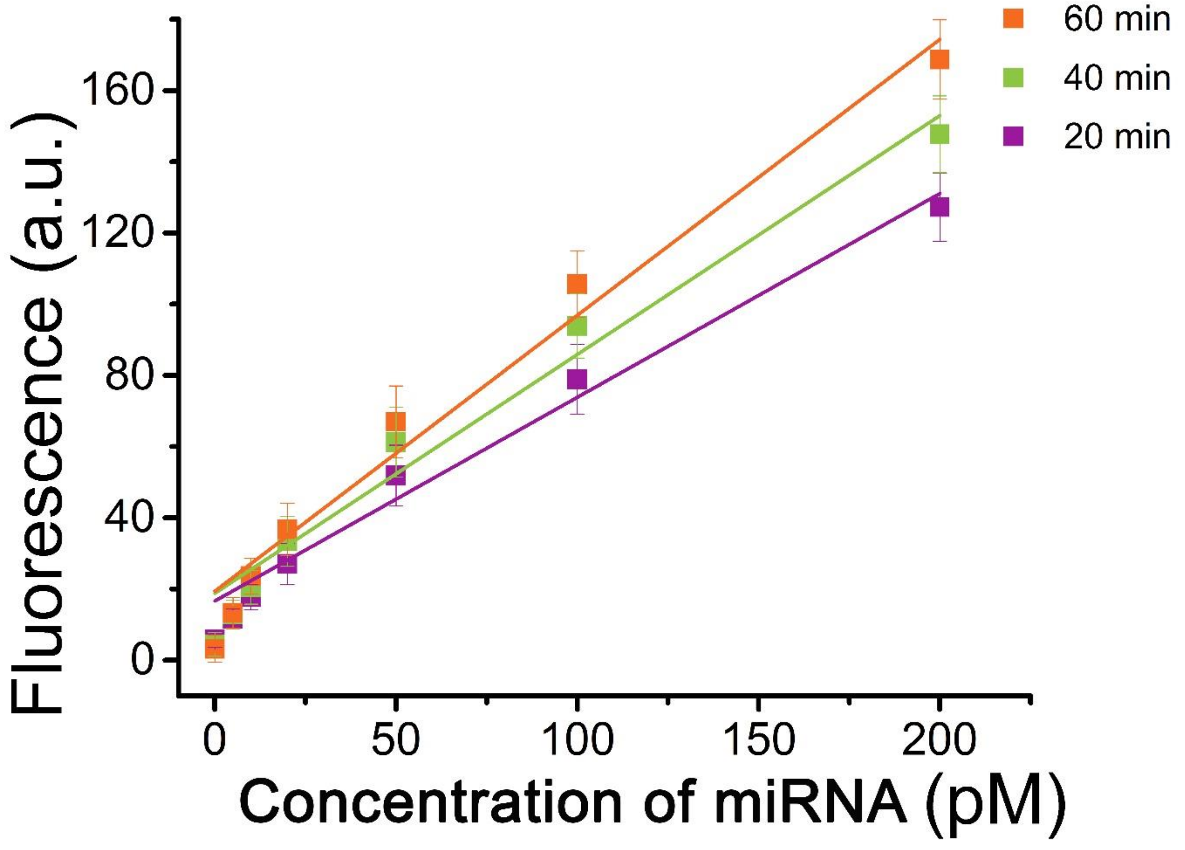


**Supplementary Figure 11**. Intensity of fluorescence generated by the DNAzyme motor in response to various concentrations of the target miRNA. The fluorescence intensity (arbitrary unit) was measured at 20, 40, and 60 min after the activation by the cofactor $Mn^{2+}$. Error bars represent one standard deviation from duplicate experiments.

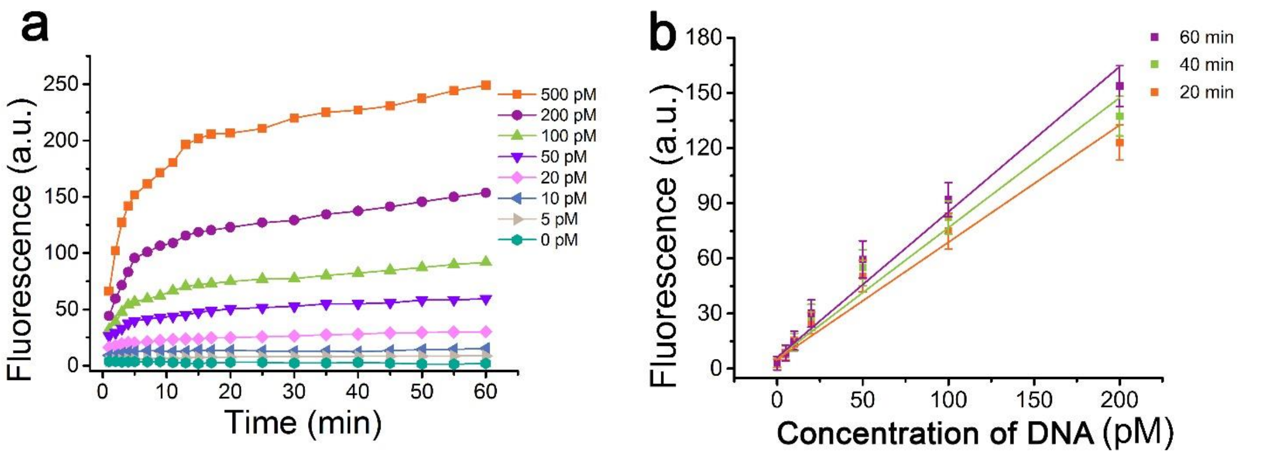


**Supplementary Figure 12.** Intensity of fluorescence generated by the DNAzyme motor in response to varying concentrations (0-500 pM) of the target DNA. Duplicate experiments were carried out for all tests and relative standard deviations (RSDs) were in the range of 2.7-8.6%.

(a) Real-time monitoring of fluorescence generated by the DNAzyme motor in response to varying concentrations (0-500 pM) of the target DNA. Time 0 refers to when the cofactor $Mn^{2+}$ was added to activate the motor.

(b) Relationships between the concentration of the target DNA and fluorescence intensity (arbitrary unit) measured at 20, 40, and 60 min after the addition of the cofactor $Mn^{2+}$. Error bars represent one standard deviation from duplicate experiments.

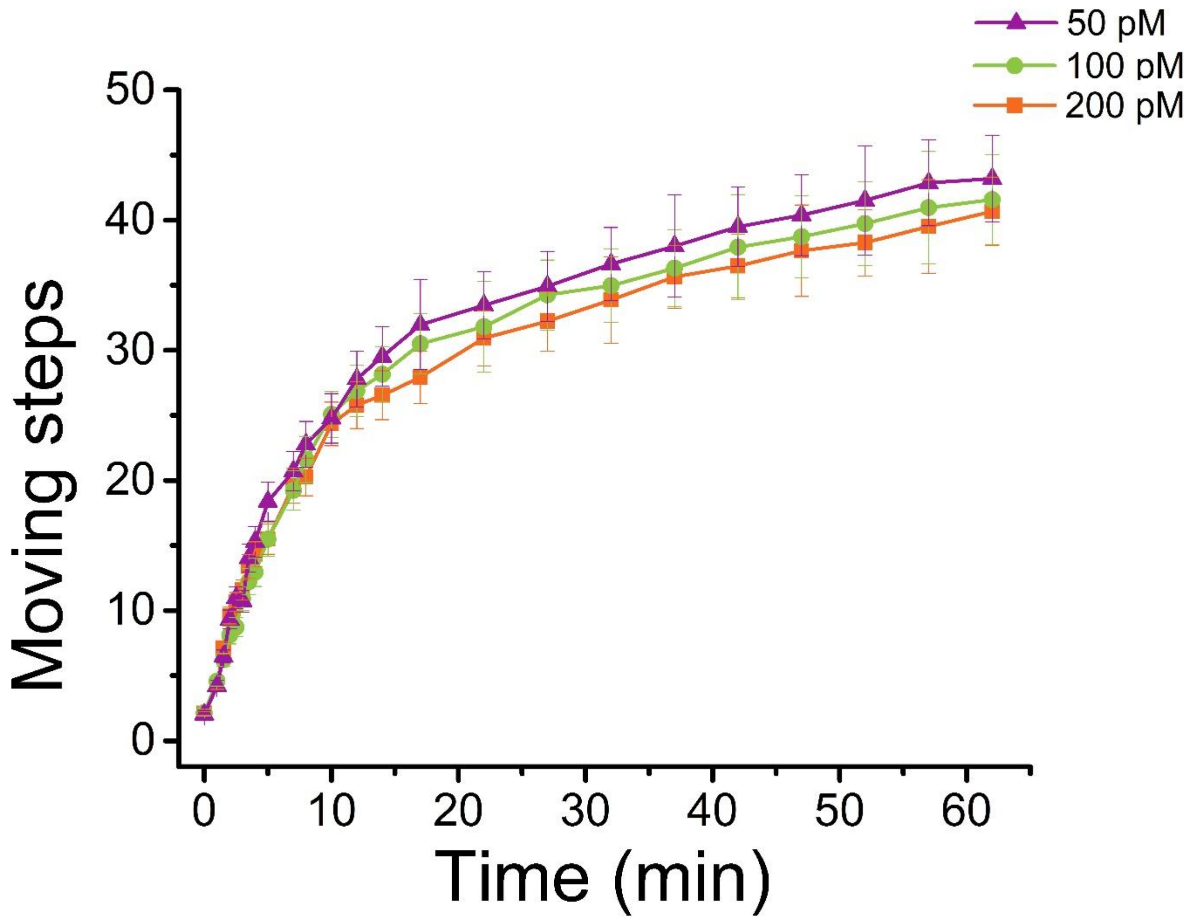


**Supplementary Figure 13**. Moving steps of DNAzyme motors, initiated by 50, 100, and 200 pM target miRNA. Error bars represent one standard deviation from duplicate experiments.

The moving steps were estimated from measuring the fluorescence of the cleaved fluorescent substrate (**F1**). The concentration of the fluorescent substrate segment **F1** was determined against a calibration curve that was constructed by using standard solutions of FAM-labeled substrate. For example, from the calibration, the concentration of the fluorescent substrate segment **F1** after 27 min of the motor operation was determined to be 6.2 nM. This was initiated by 200 pM target miRNA. Because the concentration of miRNA is lower than that of the DNAzyme motor, each target miRNA molecule activates a single DNAzyme motor. Each walking step of the motor generates a substrate segment **F1**. Therefore, the detected overall 6.2 nM substrate segment **F1** is a result of 31 average walking steps of each DNAzyme motor initiated by 200 pM (0.2 nM) target miRNA (6.2 nM/0.2 nM = 31).

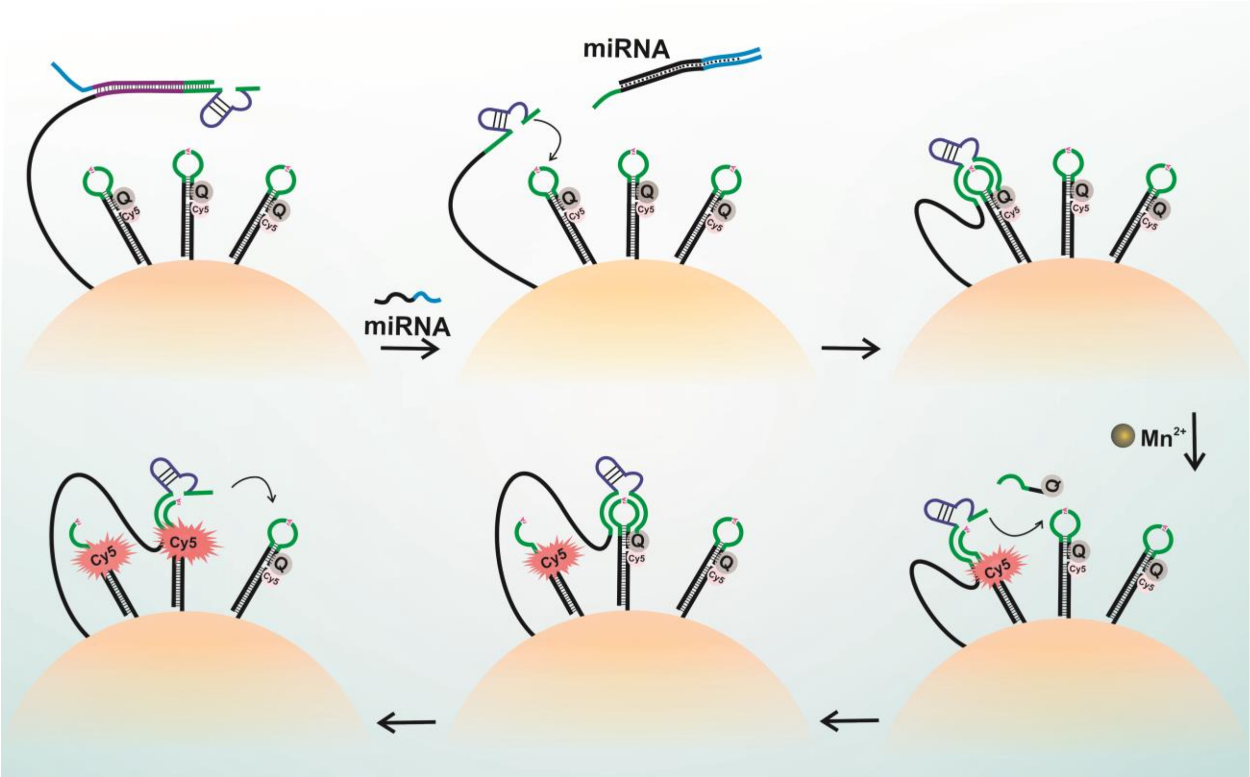


**Supplementary Figure 14.** Schematic showing the operation of the DNAzyme motor designed to trace the walking of the DNAzyme motor on individual AuNPs. The substrate strand is designed to enable each walking step of the motor to turn on the fluorescence of a Cy5 molecule on the AuNP. The substrate strand contains a hairpin structure with a long single-stranded overhang at the 5'-end that hybridizes to a Cy5-labeled DNA strand. The 3'-end of the substrate strand is labeled with a black hole quencher so that hybridization of the substrate strand to the Cy5-labeled strand quenches the fluorescence of Cy5 by the quencher in close proximity. The motor system is constructed by conjugating each AuNP with dozens of locked DNAzyme strands and hundreds of hybrids between the hairpin substrate and the Cy5-labeled strands. AuNPs (50 nm) are used to construct the motor system for practical detection by total internal reflection fluorescence microscopy (TIRFM). In the presence of the target miRNA sequence, the locked DNAzyme is activated to cleave the substrate at the single-ribonucleotide junction in the hairpin loop. The cleavage disrupts the hairpin structure and releases a quencher-containing fragment from the AuNP, restoring the fluorescence of the Cy5 molecule. The DNAzyme dissociates from the cleaved substrate and hybridizes to the next substrate strand, enabling the walking of the DNAzyme motor from one substrate strand to the next. Each walking step restores the fluorescence of one Cy5 molecule that is attached onto the AuNP through a double-stranded DNA between the substrate overhang and the Cy5-labeled strand. Quenching of Cy5 fluorescence by AuNP is moderate because of the double-stranded DNA between Cy5 and AuNP. The estimated distance from Cy5 to the surface of the AuNP is about 11 nm (32 bp). Therefore, operation of the DNAzyme motor on individual AuNPs can be traced by measuring the fluorescence increase of each AuNP.

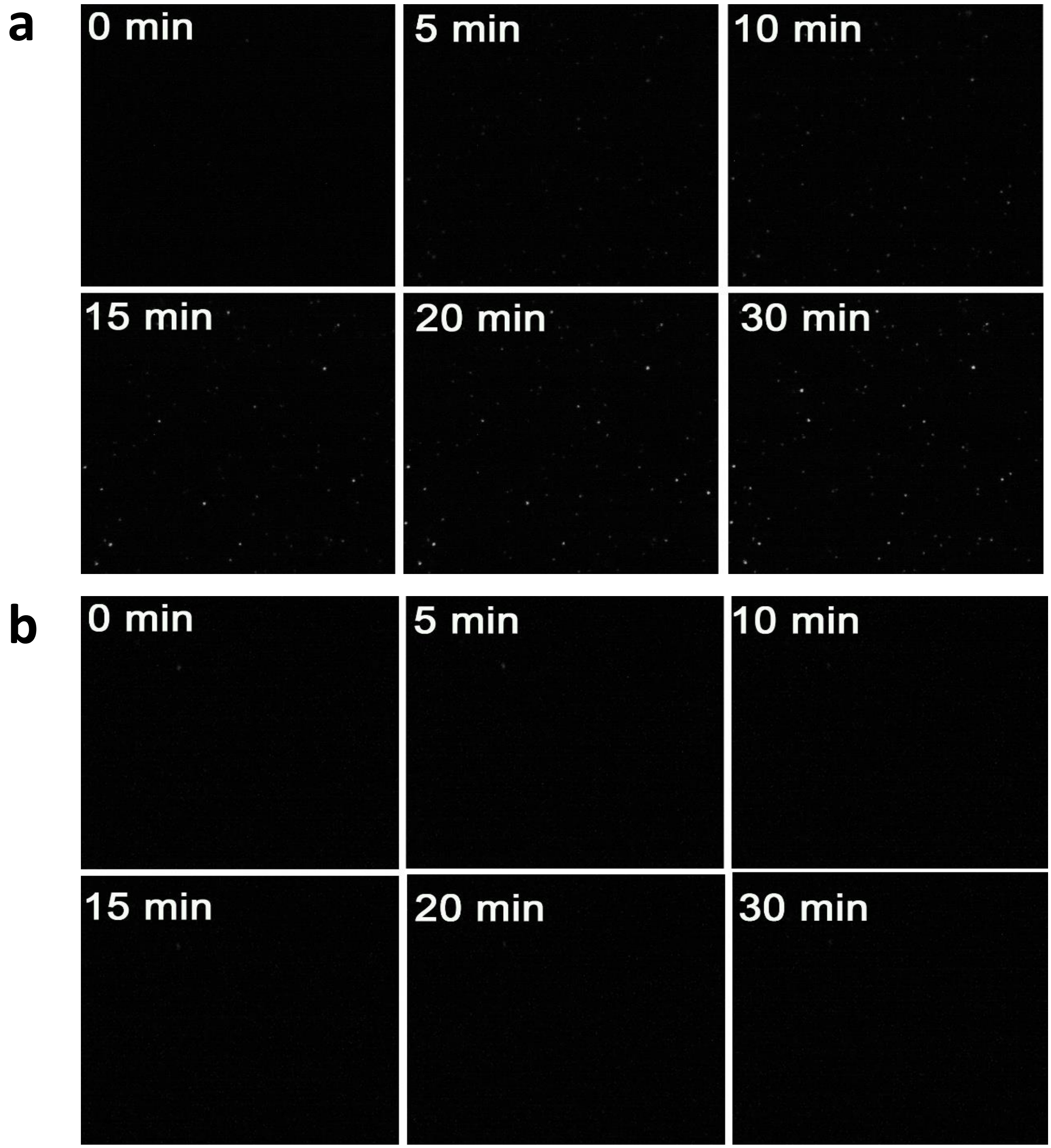


**Supplementary Figure 15**

**Supplementary Figure 15.** Fluorescence images showing operation of the DNAzyme motor on individual AuNPs in the presence (a) or absence (b) of target miRNA sequence. The frames were extracted from Supplementary Movie 1a (a) and Supplementary Movie 1b (b). The white dots on black background in (a) are due to fluorescent Cy5 on the AuNPs as depicted in Supplementary Fig. 14.

Forty five microliters of the operating solutions contained either 300 pM target miRNA (a) or no target miRNA (b), the DNAzyme motor (Supplementary Fig. 14) at an equivalent concentration of 30 pM functionalized AuNP, 25 mM Tris-acetate buffer (pH 8.0), and 200 mM NaCl. After incubation at room temperature for 20 min, 0.5 mM $MnCl_2$ solution was added to initiate the operation of the motor. The time 0 min in the figure refers to when the $MnCl_2$ was added. One μL of the incubation mixture was transferred to a microscope slide (25×76×1.0 mm; Fisher). A micro coverglass (diameter: 18 mm, thickness: 0.16-0.19 mm; Electron Microscopy Sciences) was placed over the solution on top of the slide. The AuNPs sandwiched between the microscope slide and the coverglass were then imaged using a DeltaVision OMX Imaging System (GE Healthcare Life Sciences). A 60X/1.49 TIRF objective (Nikon) was used, and a 647-nm laser provided excitation. One hundred and twenty frames were acquired in 30 min with 10% laser power and 100 ms exposure time for each frame.

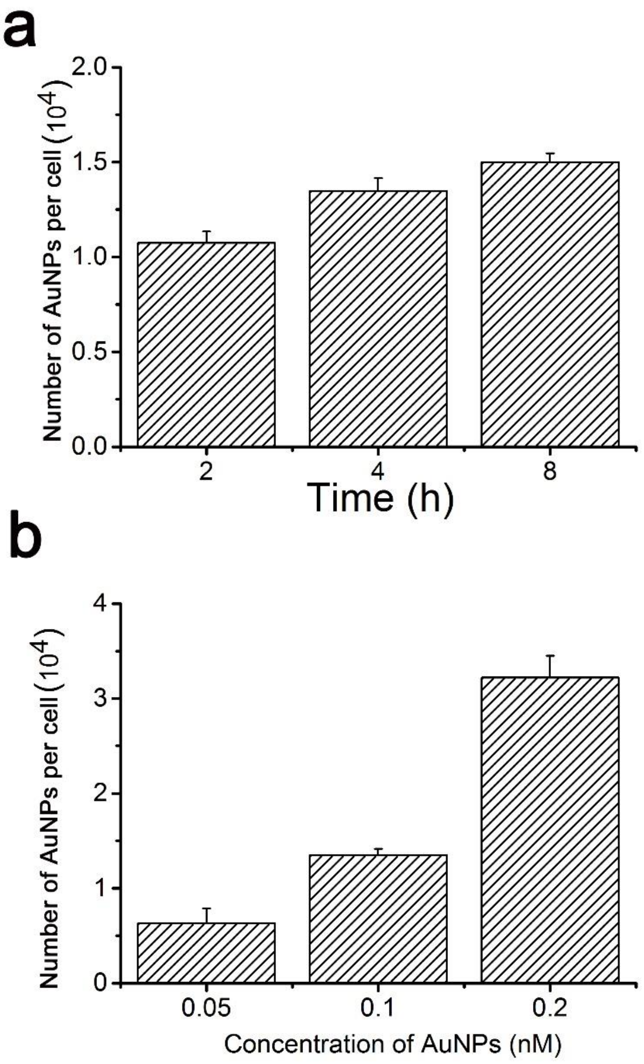


**Supplementary Figure 16**. Uptake of DNAzyme motor system into MDA-MB-231 cells. The concentrations of Au in the cells were measured by using inductively coupled plasma mass spectrometry (ICP-MS). (a) Effect of the incubation time. (b) Effect of the incubation concentration of AuNPs on which the DNAzyme motor system is constructed. Error bars represent one standard deviation from triplicate experiments.

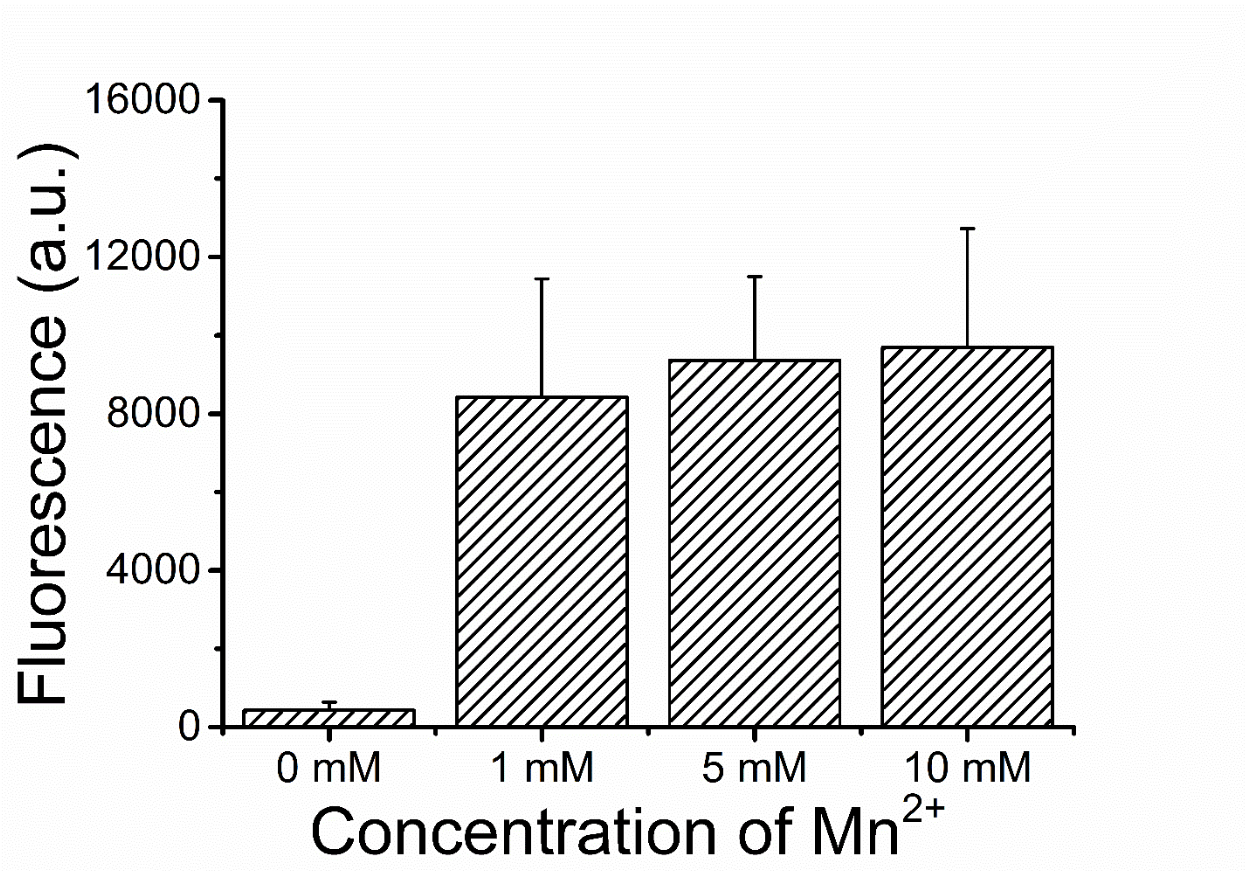


**Supplementary Figure 17**. Effect of treatment concentration of $Mn^{2+}$ on the intracellular operation of the DNAzyme motor. The fluorescence intensity was determined from fluorescence images of cells 60 min after the addition of $Mn^{2+}$. The measurement of fluorescence was carried out using ImageJ 1.47. Error bars represent one standard deviation from triplicate experiments.

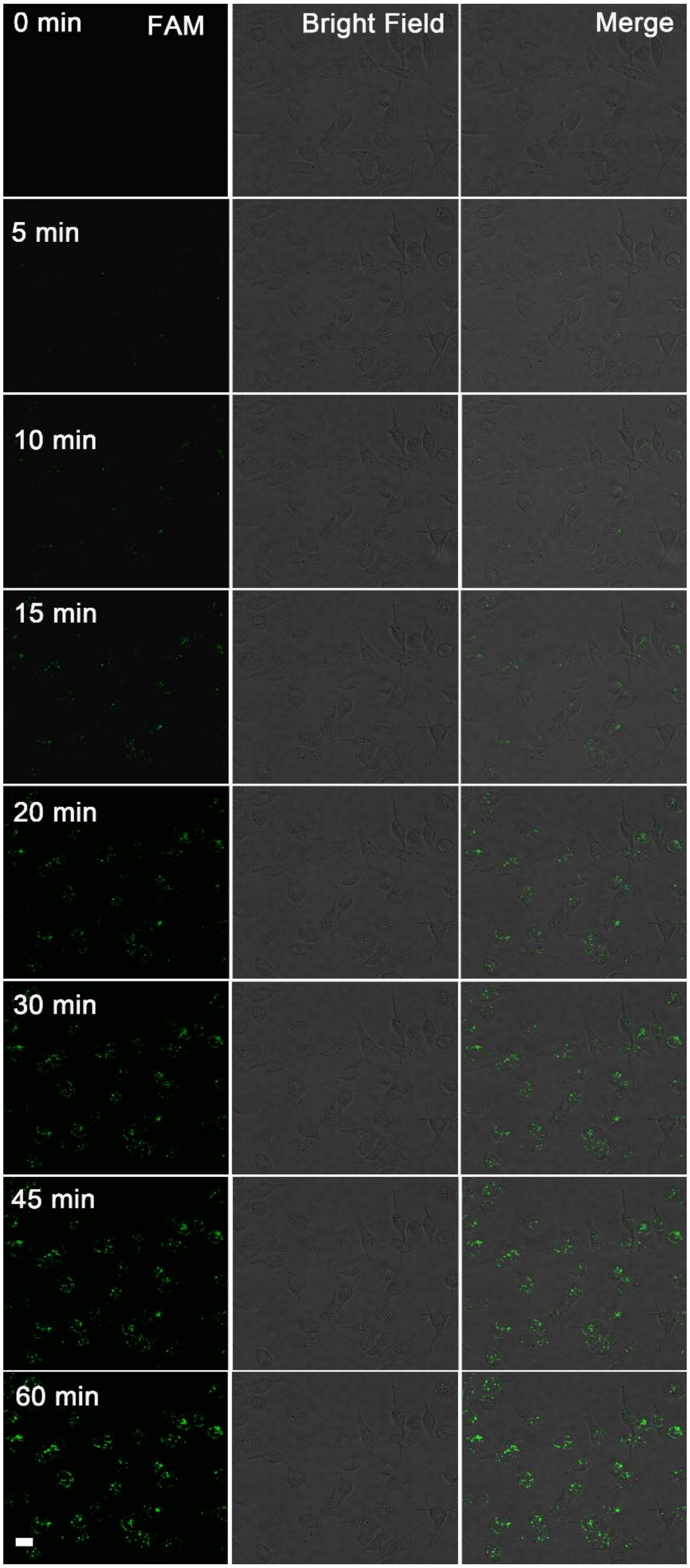


**Supplementary Figure 18**

**Supplementary Figure 18**. Images of MDA-MB-231 cancer cells following intracellular operation of the DNAzyme motor. These images are representative frames from the Supplementary Movie 2. MDA-MB-231 cancer cells were incubated with the DNAzyme motor for 2 h. The cells were washed and then $Mn^{2+}$ and the operating buffer were added. Immediately after the addition of $Mn^{2+}$, fluorescence images of the cells were repeatedly acquired for 60 min at a rate of 2 frames per min. The observed fluorescence in the cells is a result of the intracellular operation of the DNAzyme motor initialed by the target microRNA (miR-10b) in the cells and activated by the cofactor $Mn^{2+}$. An Olympus IX-81 fluorescence microscope coupled with a Yokagawa CSU × 1 spinning disk confocal scan-head was used. The LMM5 laser transmission setting was 20 and the laser excitation time was 495 ms for each frame of the fluorescence images. The length of the scale bar is 17 μm.

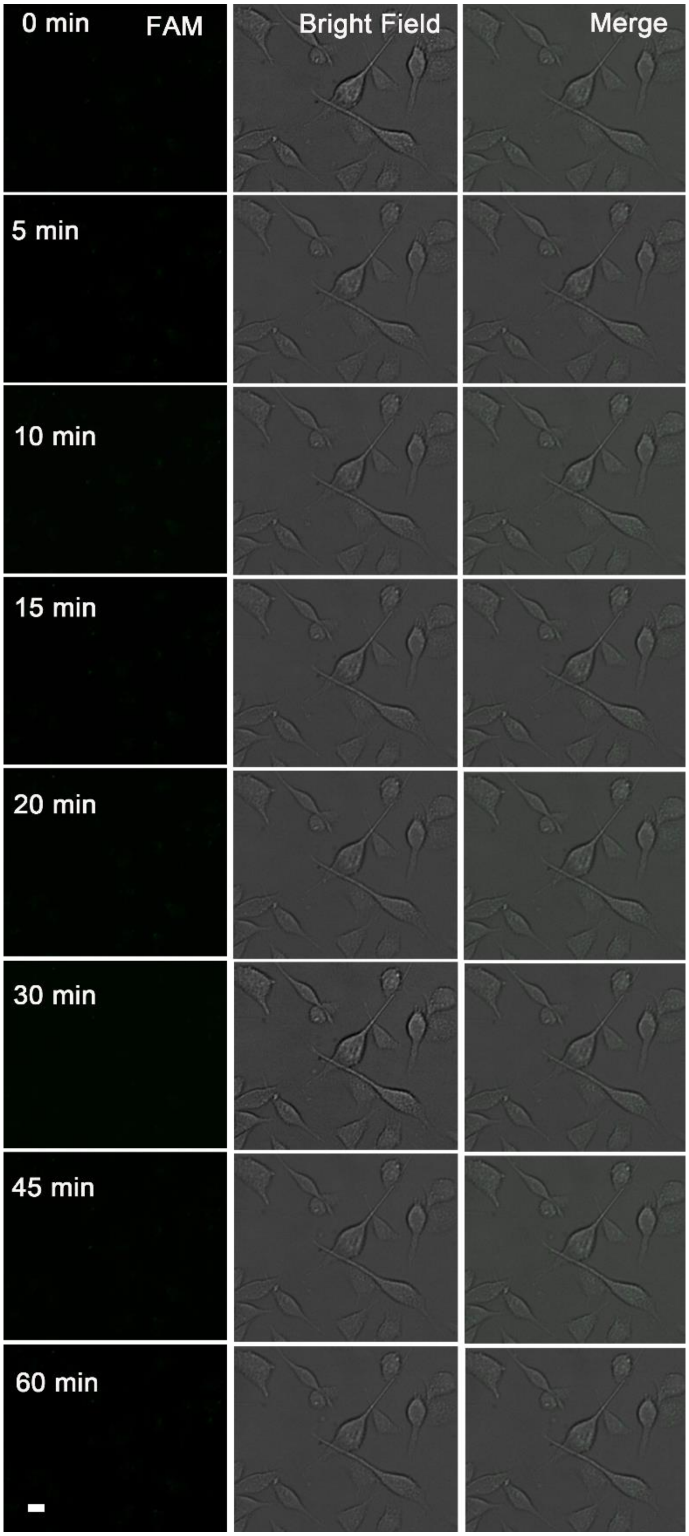


**Supplementary Figure 19**. Images of MDA-MB-231 cancer cells after incubation with the mutant DNAzyme motor for 2 h followed by the addition of $Mn^{2+}$ and fluorescence imaging at 0, 5, 10, 15, 20, 30, 45, and 60 min after the addition of $Mn^{2+}$. No fluorescence is observed from the cells, indicating that the mutant DNAzyme motor is inactive in the cells. The same microscopy conditions as shown in Supplementary Fig. 18 were used. The length of the scale bar is 17 µm.

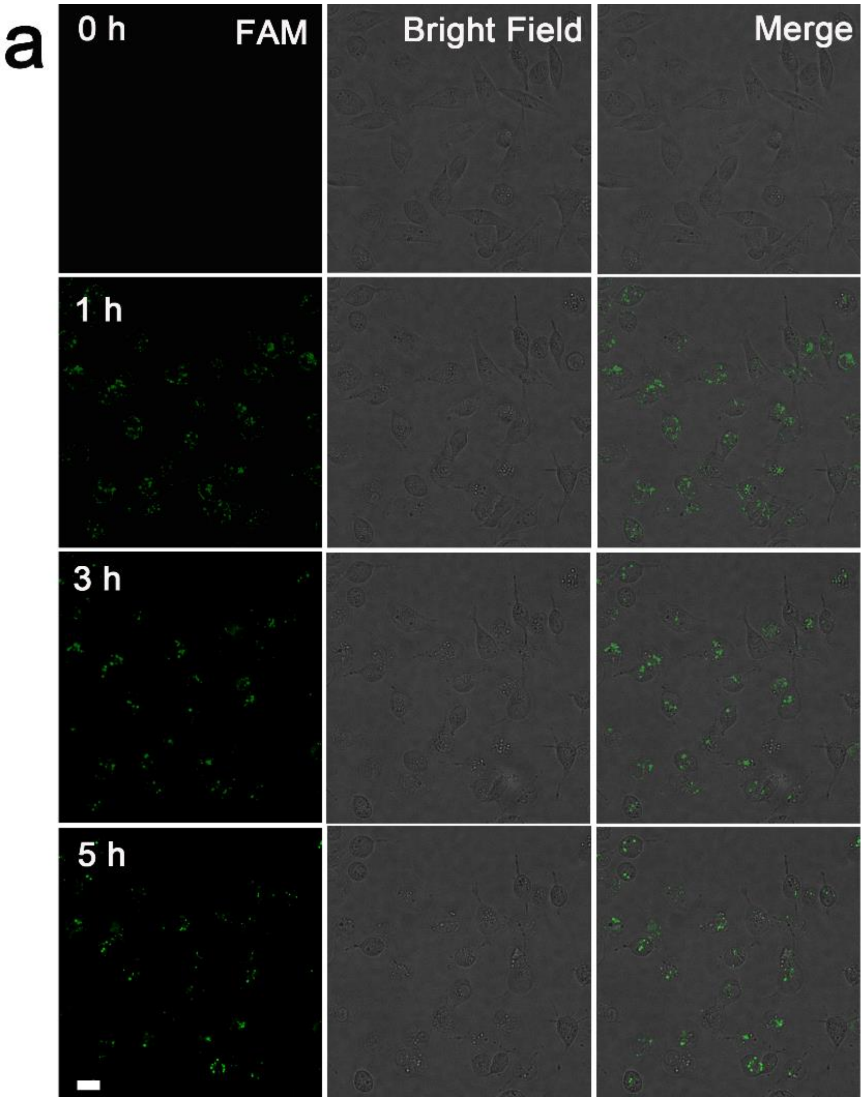


**Supplementary Figure 20a.** Images of MDA-MB-231 cancer cells after intracellular operation of the DNAzyme motor for 0, 1, 3, and 5 hours. MDA-MB-231 cancer cells were incubated with the DNAzyme motor for 2 h. The cells were washed and then $Mn^{2+}$ in the operating buffer was added. Immediately after the addition of $Mn^{2+}$, fluorescence images of the cells were repeatedly acquired over the 5-h period. The observed fluorescence in the cells is a result of the intracellular operation of the DNAzyme motor initialed by the target microRNA (miR-10b) in the cells and activated by the cofactor $Mn^{2+}$. The slight decrease of fluorescence intensity observed after 5 h is probably due to photo bleaching of the fluorescent substrate. The length of the scale bar is 17 μm.

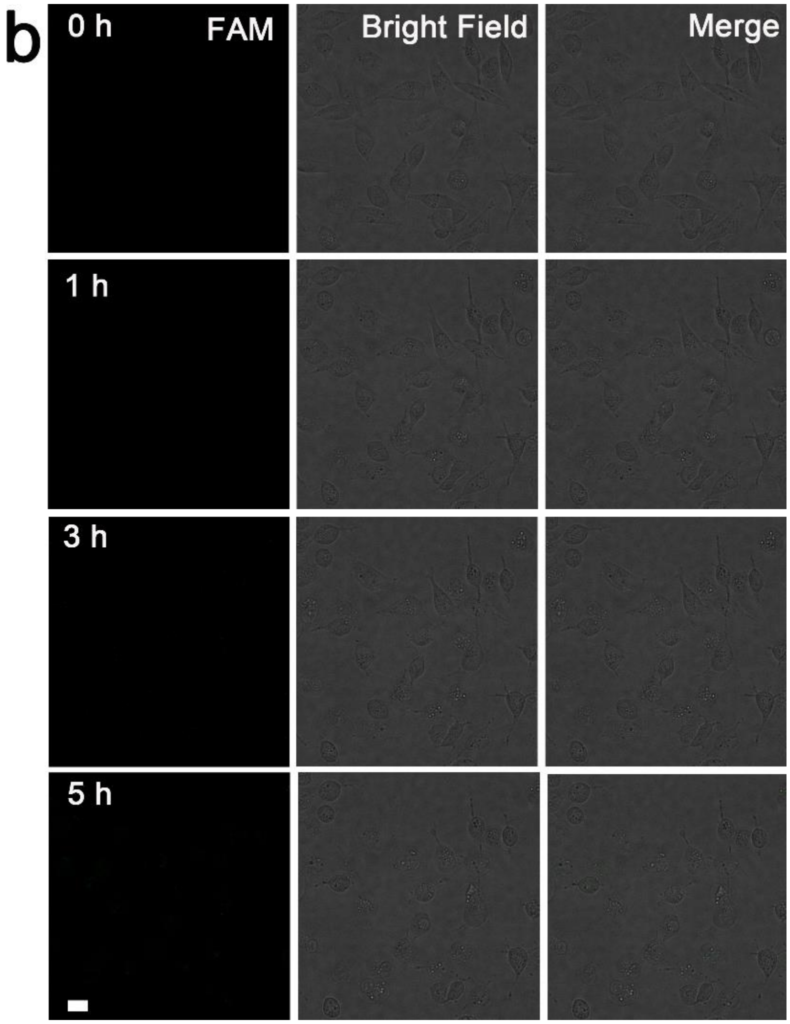


**Supplementary Figure 20b.** Images of MDA-MB-231 cancer cells after incubation with the mutant DNAzyme motor for 2 h followed by the addition of $Mn^{2+}$ and fluorescence imaging at 0, 1, 3, and 5 h after the addition of $Mn^{2+}$. No fluorescence is detectable from the cells, indicating that the substrate strands on AuNPs are stable in the cells. The length of the scale bar is 17 μm.

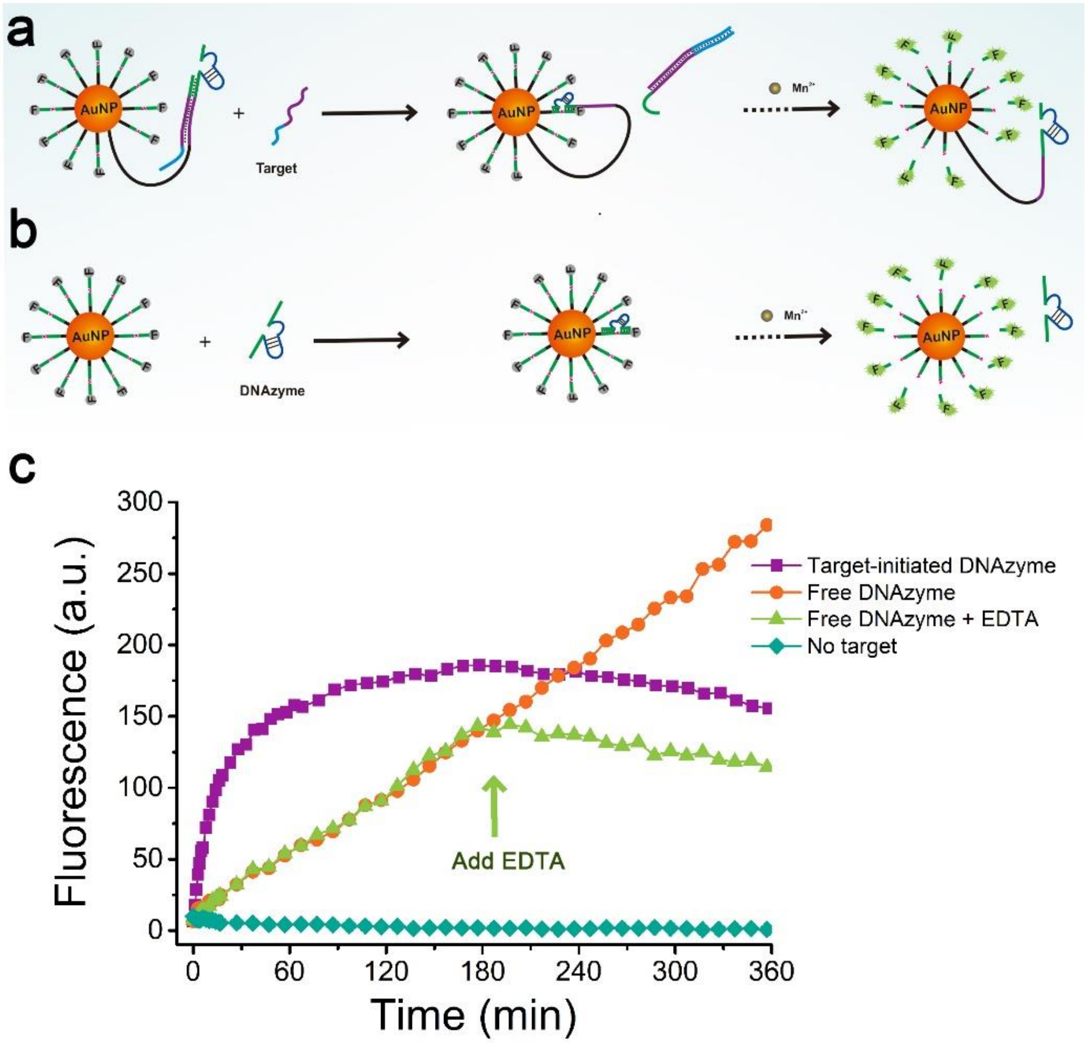


**Supplementary Figure 21**. Comparison of walking and results of catalytic cleavage of the substrate by the miRNA target-initiated DNAzyme motor and by the free control DNAzyme that is not conjugated to AuNP. (a) Scheme showing the operation of target-initiated DNAzyme motor. (b) Scheme showing the operation of the free control DNAzyme. The free control DNAzyme sequence (Supplementary Table 1) has an 8-nt **Arm 1** and an 8-nt **Arm 2** to assist its hybridization to the substrate. (c) The operating curves of the target-initiated and free DNAzyme motors. The operation of motors was monitored for 6 h following the addition of cofactor $Mn^{2+}$. Relative standard deviations from replicate measures were in the range of 1-5%.

The small decrease of fluorescence from the target-initiated DNAzyme motor after 3 h is probably due to photo bleaching by extended period of exposure to excitation light. The following experiment supports this: 3 h after the operation of the free control DNAzyme, EDTA was added to the solution to chelate the cofactor $Mn^{2+}$ and thus stop the further operation of the DNAzyme. Continued monitoring of the fluorescent substrate segment produced during the first 3 h by the free DNAzyme shows the same slight decreasing pattern as in the case of the target-initiated DNAzyme motor.

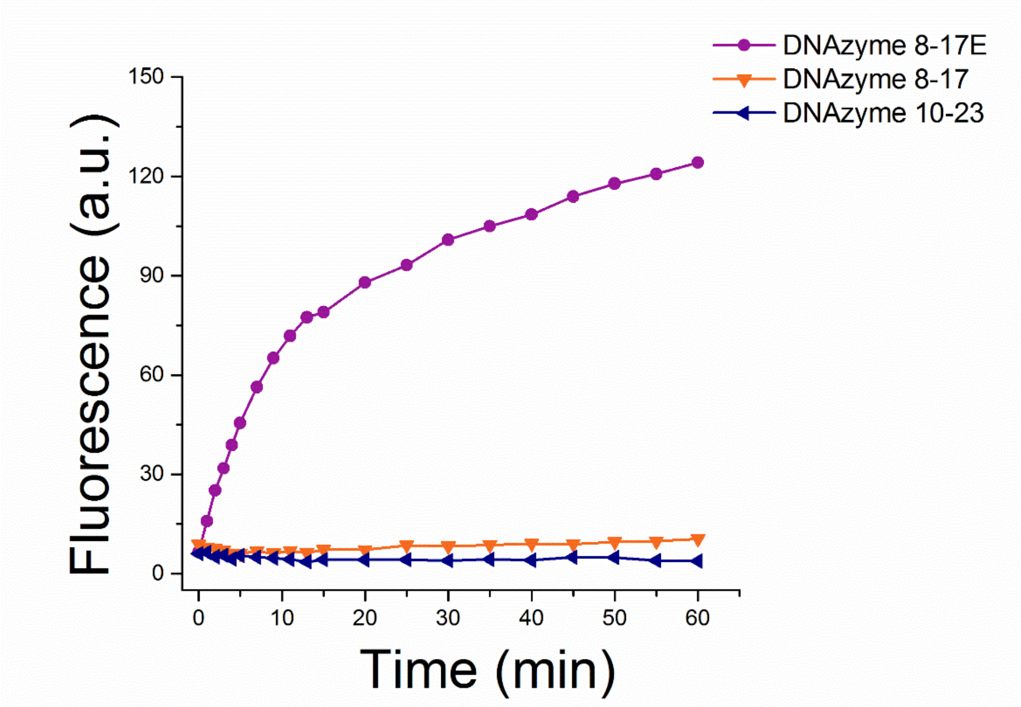


**Supplementary Figure 22**. Comparison of the operation of the motors constructed with three different DNAzymes, 10-23, 8-17, and 8-17E. These DNAzymes were biotin-labeled and their sequences are shown in Supplementary Table 1. AuNPs were conjugated with hundreds of substrate strands and dozens of biotin-labeled poly thymine (30 T). Streptavidin was then used to link the biotin-labeled DNAzyme onto the AuNPs. Specifically, operation solutions were prepared to contain 230 pM AuNPs, 200 pM streptavidin, 2 nM biotin-labeled DNAzyme strand in 25 mM Tris-acetate (8.0) and 200 mM NaCl. After incubation at room temperature for 20 min, 500μM $Mn^{2+}$ was then added to the solutions to activate the operation of the motors. The fluorescence of the solutions was then monitored in real-time for 60 min. Relative standard deviations from duplicate measures were in the range of 2.2-6.4%.

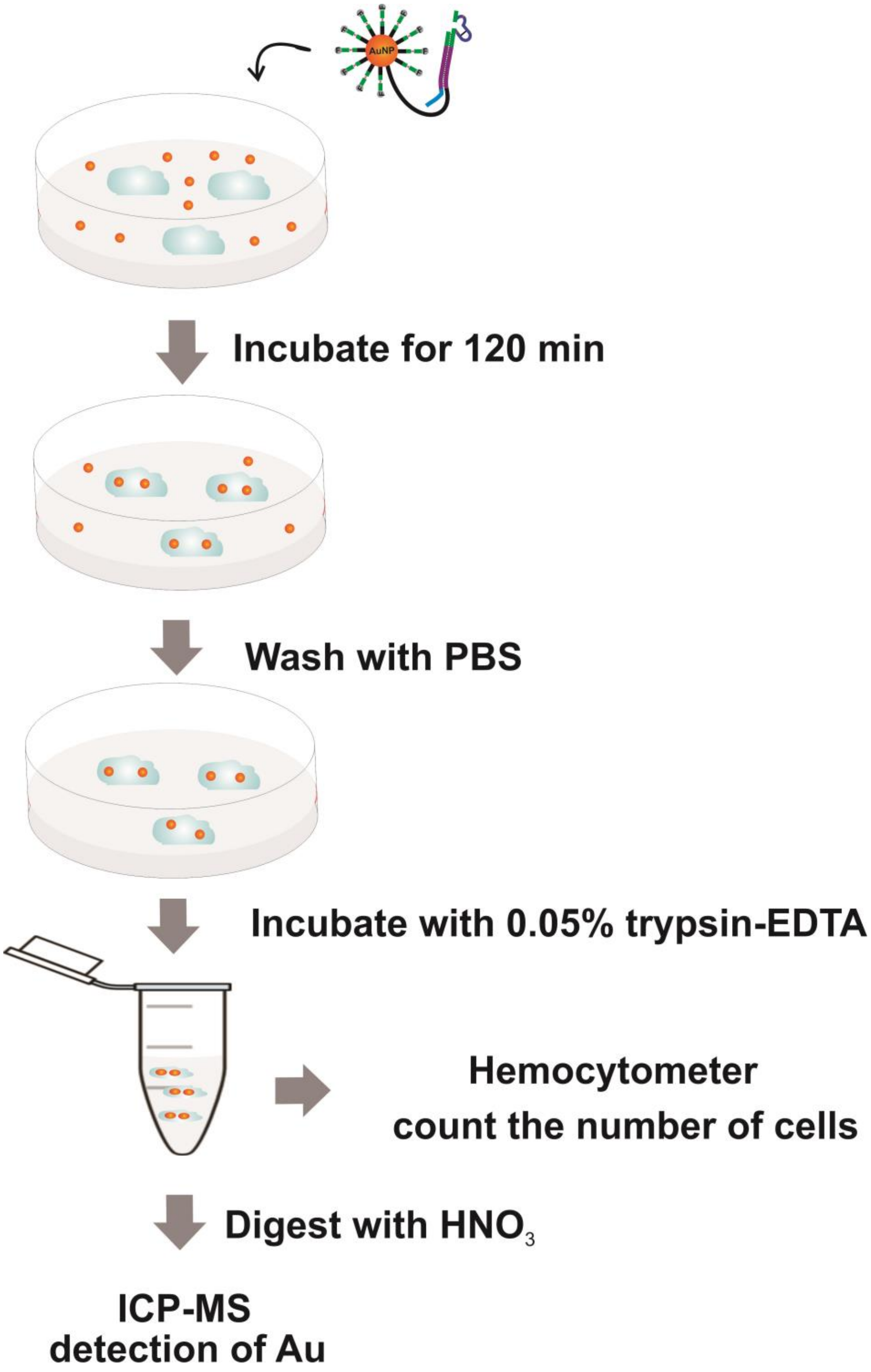


**Supplementary Figure 23.** Schematic showing the experiments conducted to determine cellular uptake of the DNAzyme motor system. The DNAzyme motor system consists of AuNPs functionalized with the substrate and the locked DNAzyme sequences.

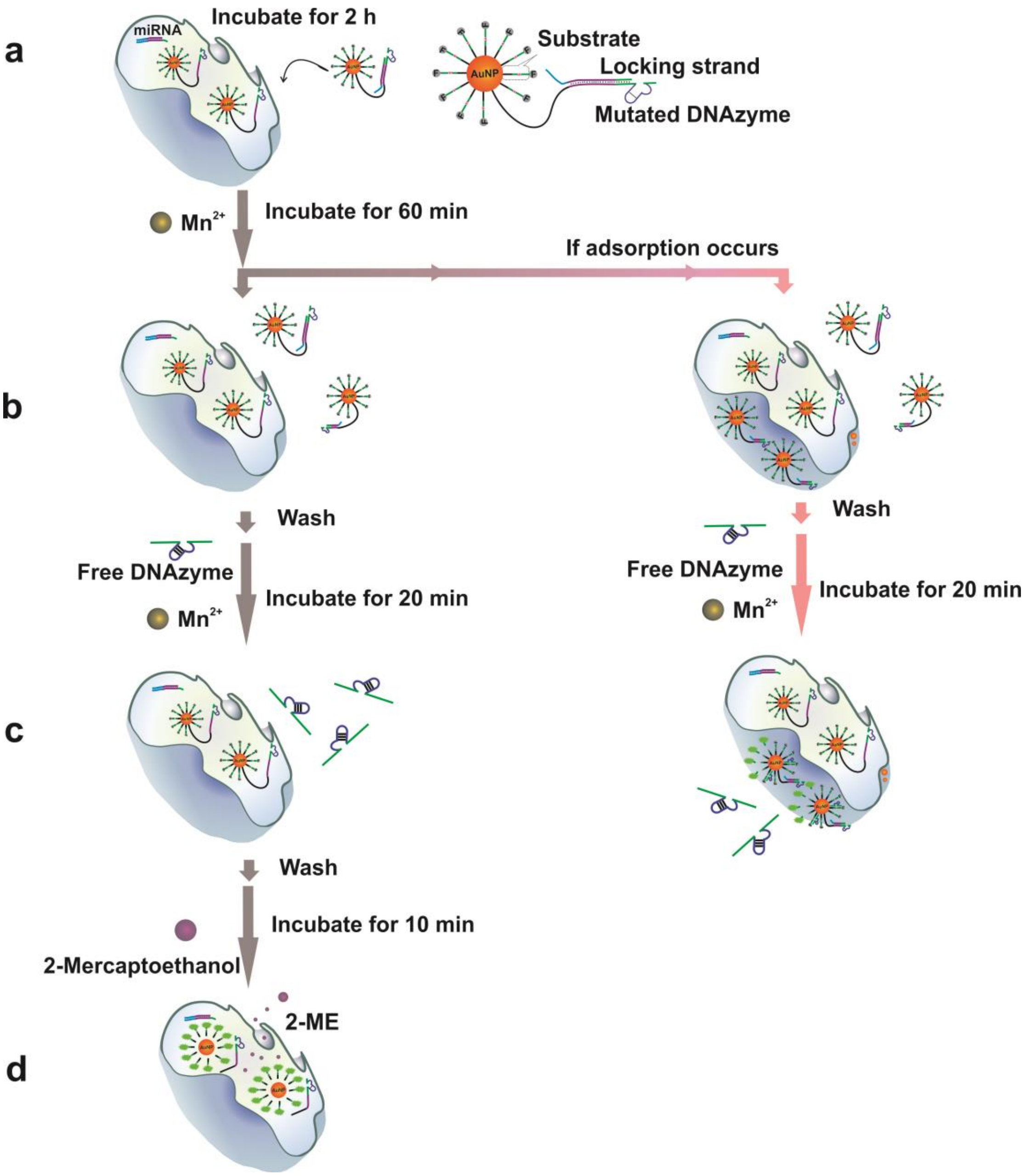


**Supplementary Figure 24.** Schematic showing the experiments conducted to test whether adsorption of the DNAzyme motor system (functionalized AuNPs) could be a confounding issue. Fluorescence from the same set of the target MDA-MB-231 cancer cells was repeatedly measured after each treatment (a, b, c, and d). The results are shown in Supplementary Fig. 25. If mutant DNAzyme motors (consisting of the functionalized AuNPs) were adsorbed on the cell surface (shown in the right-hand side portion of the schematic), the free control DNAzyme would cleave the fluorescent substrate from the AuNPs and produce fluorescence. The results in Supplementary Fig. 25c show no detectable fluorescence from the target cells, suggesting that adsorption of the DNAzyme motor system on the cell surface is negligible.

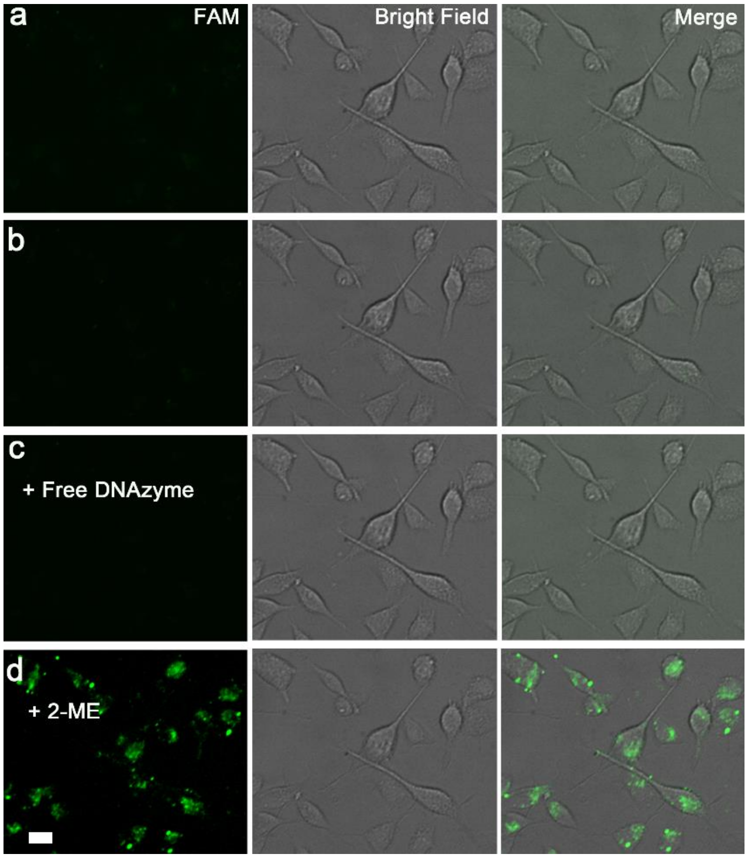


**Supplementary Figure 25.** Images showing target MDA-MB-231 cancer cells after incubation with the mutant DNAzyme motor. (a) Images of MDA-MB-231 cancer cells after incubation with the mutant DNAzyme motor for 2 h. (b) Images of the cells after further incubation in the presence of $Mn^{2+}$ cofactor. Little fluorescence was observed, suggesting that the mutant DNAzyme motor is inactive. (c) Images of the same MDA-MB-231 cancer cells after further incubation with 200 pM free control DNAzyme for 20 min. Little fluorescence is observed from the target cells, suggesting that adsorption of the DNAzyme motor system on the cell surface is negligible. (d) Images of the MDA-MB-231 cancer cells from (c) after additional treatment with 10 mM 2-mercaptoethanol (2-ME). Strong fluorescence is observed, suggesting that AuNPs of the mutant DNAzyme motor system have entered the target cells. The experimental procedures are shown in Supplementary Fig. 24. The length of the scale bar is 17 μm.

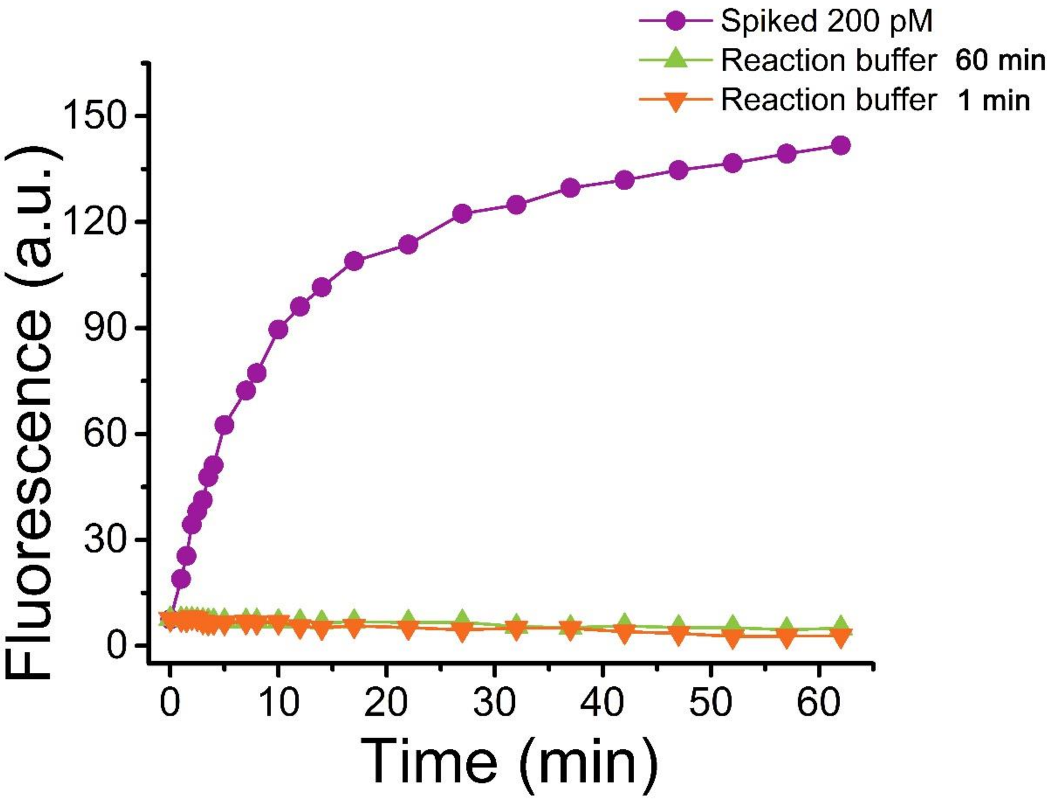


**Supplementary Figure 26.** Real-time monitoring of fluorescence generated by the DNAzyme motor in the reaction buffer after separation of the target cells.

These experiments were designed to test whether the target miRNA could leak out of the cells and then initiate operation of the DNAzyme motor outside of the cells. A DNAzyme reaction buffer, containing 25 mM Tris-acetate (pH 8.0) and 125 mM NaCl, was added to the MDA-MB-231 cells, and this reaction buffer solution was removed either 1 h after incubation with the cells or immediately after its contact with the cells (1 min). The DNAzyme motor system at an equivalent AuNP concentration of 230 pM and 0.5 mM $MnCl_2$ were added to these reaction buffer solutions. Fluorescence was monitored for 60 min. If the target miRNA had leaked out of the cells, then the target miRNA in this solution would initiate the operation of the DNAzyme motor and produce fluorescent substrate. However, no fluorescence increase is observed, suggesting that very little target miRNA is present outside of the cells. As a positive control, further addition of 200 pM target miRNA into the solution results in an expected fluorescence increase. Relative standard deviations from duplicate measurements were 4.1-8.4%.
